# Huperzine A Attenuates Oxidative Stress-Induced H9c2 Cardiomyoblast Injury Through AKT-Associated Survival Signaling

**DOI:** 10.3390/antiox15101333

**Published:** 2026-10-15

**Authors:** Yusong Zhang, Hongxia Long, Li Qiao, Huiling Zhang, Dingyuan Chen, Jingjing Chen, Bo Wei, Wei Li, Haiyan Zhou

**Affiliations:** 1 Department of Cardiovascular Disease, The Affiliated Hospital of Guizhou Medical University, Guiyang 550000, China; 2 Department of Clinical Medicine, Guizhou Medical University, Guiyang 550000, China; 3 Department of Cardiovascular Disease, Anshun People’s Hospital, Anshun 561000, China; 4 Department of Clinical Research Centre, The Affiliated Hospital of Guizhou Medical University, Guiyang 550000, China

**Keywords:** Huperzine A, oxidative stress, cardiomyoblast injury, AKT, MCL-1, HMGB1

## Abstract

Oxidative stress is a major contributor to cardiomyoblast injury and cardiovascular disease progression. Huperzine A (HupA), a natural Lycopodium alkaloid, exhibits antioxidant and anti-inflammatory properties, but its cardioprotective mechanisms remain unclear. This study aimed to investigate the effects of HupA on oxidative stress-induced H9c2 cardiomyoblast injury and elucidate the underlying molecular mechanisms. Network pharmacology, molecular docking, molecular dynamics simulations, and cellular thermal shift assay (CETSA) were integrated to predict and investigate AKT1 involvement. An in vitro model of tert-butyl hydroperoxide (tBHP)-induced injury in H9c2 rat cardiomyoblasts was established. Oxidative stress status, mitochondrial function, and AKT-associated signaling were evaluated. The involvement of AKT signaling was examined using MK-2206. HupA significantly improved cell viability in a dose-dependent manner and markedly reduced lipid peroxidation and intracellular and mitochondrial ROS accumulation. In addition, HupA restored antioxidant defenses by increasing superoxide dismutase activity and glutathione levels. Mitochondrial dysfunction induced by tBHP was effectively alleviated, as evidenced by preserved mitochondrial membrane potential and reduced mitochondrial superoxide production. Furthermore, HupA enhanced AKT phosphorylation and MCL-1 expression while inhibiting extracellular HMGB1 release. Importantly, pharmacological inhibition of AKT largely abolished these protective effects, including antioxidant restoration, mitochondrial preservation, and anti-cell death activity. HupA protects H9c2 cardiomyoblasts against oxidative injury through AKT-associated survival signaling, thereby attenuating oxidative stress and preserving mitochondrial integrity. These findings suggest HupA as a promising therapeutic candidate in H9c2 cardiomyoblasts under oxidative stress conditions.

## 1. Introduction

Cardiovascular diseases remain the leading cause of morbidity and mortality worldwide, with myocardial injury representing a central pathological process in conditions such as acute myocardial infarction, ischemia–reperfusion injury, heart failure, and cardiotoxicity [1,2,3]. Regardless of the initiating stimulus, excessive oxidative stress is recognized as one of the principal mechanisms driving cardiomyoblast dysfunction and death. During oxidative stress, excessive production of reactive oxygen species (ROS) overwhelms endogenous antioxidant defenses [4], leading to lipid peroxidation, impaired mitochondrial oxidative phosphorylation (OXPHOS) [5], mitochondrial dysfunction [6], DNA damage [7], and activation of cell death pathways [8]. Therefore, identification of effective antioxidant strategies to preserve mitochondrial function and limit oxidative damage has become an important therapeutic objective for myocardial injury [9,10].

Mitochondria are both the major source and primary target of intracellular ROS. Oxidative stress-induced mitochondrial dysfunction results in dissipation of mitochondrial membrane potential (ΔΨm) [11], excessive mitochondrial ROS generation, ATP depletion, and release of damage-associated molecular patterns (DAMPs), thereby amplifying inflammatory responses and cardiomyoblast death [12]. Among the signaling pathways regulating mitochondrial homeostasis, the phosphatidylinositol 3-kinase/protein kinase B (PI3K/AKT) pathway plays a critical role in promoting cell survival and resistance to oxidative injury [13]. Activated AKT1 enhances the expression of anti-apoptotic proteins such as myeloid cell leukemia-1 (MCL-1), preserves mitochondrial integrity, and suppresses oxidative stress-induced cell death [14]. In cardiomyoblasts, previous studies have also suggested that PI3K/AKT signaling can regulate HMGB1 expression under pathological stress conditions [15]. Moreover, mitochondrial dysfunction promotes the extracellular release of high mobility group box 1 (HMGB1), an important DAMP that aggravates inflammation and tissue injury [16]. These observations suggest that AKT1-dependent survival signaling may be functionally interconnected during oxidative injury.

Huperzine A (HupA), a naturally occurring Lycopodium alkaloid isolated from Huperzia serrata, has been extensively investigated for its neuroprotective properties and is clinically used for the treatment of Alzheimer’s disease because of its potent acetylcholinesterase inhibitory activity [17,18]. Beyond its neurological effects, experimental studies have reported antioxidant, anti-inflammatory, and anti-apoptotic properties of HupA in several cellular and animal models. These pharmacological activities, particularly its reported effects on oxidative stress and mitochondrial function, provide a rationale for investigating its potential effects in other cell types that are highly sensitive to oxidative injury [19,20,21]. Importantly, emerging evidence also suggests that HupA may exert cardioprotective effects. A sustained-release formulation of HupA, HupA microspheres (HAM), was reported to attenuate isoprenaline-induced myocardial injury in mice, as evidenced by reduced serum CK-MB and Tn-I levels, improved myocardial histopathology, and decreased circulating TNF-α and IL-6 levels [22]. However, although this finding provides evidence for a cardioprotective role of HupA, the cardiovascular actions of HupA remain incompletely characterized, particularly with respect to intracellular survival signaling in cardiomyoblasts. It remains unclear whether HupA modulates AKT-associated survival signaling in cardiomyoblasts and whether such signaling contributes to its protective effects against oxidative stress-induced cellular injury. Therefore, defining the potential relationship between HupA and AKT signaling may provide further insight into the molecular basis of its cytoprotective effects.

In the present study, an integrated strategy combining network pharmacology, molecular docking, molecular dynamics simulation, cellular thermal shift assay (CETSA) and experimental validation was employed to investigate the potential mechanisms underlying the effects of HupA under oxidative stress conditions. A tert-butyl hydroperoxide (tBHP)-induced H9c2 cardiomyoblast injury model was established to evaluate the effects of HupA on cell viability, oxidative stress, mitochondrial function, and AKT-associated signaling. Furthermore, the role of AKT signaling was examined using the selective AKT inhibitor MK-2206. Based on previous evidence, we hypothesized that AKT signaling contributes to the cytoprotective effects of HupA against oxidative stress-induced H9c2 cardiomyoblast injury.

## 2. Materials and Methods

### 2.1. Network Pharmacology Analysis

#### 2.1.1. Prediction of Huperzine A Targets

The chemical structure of Huperzine A (HupA) was obtained from the PubChem database. Potential protein targets of HupA were predicted using the SwissTargetPrediction platform with the species restricted to Homo sapiens. All predicted targets were standardized to official gene symbols using the UniProt database, and duplicate entries were removed.

#### 2.1.2. Identification of Myocardial Injury-Related Targets

Disease-related targets were collected from the GeneCards, Comparative Toxicogenomics Database (CTD), and Online Mendelian Inheritance in Man (OMIM) databases using the keywords myocardial injury, heart injury, cardiac injury, myocardial ischemia–reperfusion injury, and heart failure. After merging the datasets, duplicate genes were removed to generate the myocardial injury-related target set.

#### 2.1.3. Construction of the Drug–Disease Network

The overlapping targets between HupA and myocardial injury were identified using the Venny online platform. The common targets were imported into Cytoscape 3.9.1 to construct the compound–target interaction network.

#### 2.1.4. Protein–Protein Interaction Network and Functional Enrichment Analysis

The common targets were submitted to the STRING database for protein–protein interaction (PPI) analysis with the species limited to Homo sapiens. Hub genes were identified according to network topological parameters using Cytoscape. Gene Ontology (GO) and Kyoto Encyclopedia of Genes and Genomes (KEGG) enrichment analyses were performed using DAVID and Metascape. Enriched biological processes and signaling pathways with *p* < 0.05 were considered statistically significant.

### 2.2. Molecular Docking

The crystal structures of AKT1 (PDB ID: 8UW7), AKT2 (PDB: 8Q61), GSK3β (PDB ID: 1I09), and SRC (PDB ID: 6ATE) were downloaded from the RCSB Protein Data Bank, whereas the three-dimensional structure of HupA was obtained from PubChem. Protein structures were prepared using PyMOL3.0.3 by removing water molecules and native ligands, followed by hydrogen atom addition. Molecular docking was carried out using AutoDock Vina v1.2.5. The conformation with the lowest binding free energy was selected as the optimal binding mode and visualized using PyMOL.

### 2.3. Molecular Dynamics Simulation

To further evaluate the stability of the HupA–AKT1 complex, a 100 ns all-atom molecular dynamics (MD) simulation was performed using GROMACS 2022. The Amber14SB force field was applied to the protein, while GAFF2 parameters were assigned to HupA. Following energy minimization, NVT and NPT equilibration were performed before the production simulation.

Trajectory analyses included the root mean square deviation (RMSD), root mean square fluctuation (RMSF), radius of gyration (Rg), solvent-accessible surface area (SASA), hydrogen bond analysis, principal component analysis (PCA), free energy landscape (FEL), and MM/PBSA binding free energy calculations. The trajectory-derived parameters were analyzed and visualized using DuIvyTools 0.4.6 (https://pypi.org/project/DuIvyTools/, accessed on 21 June 2026).

The binding free energy of the HupA–AKT1 complex was estimated using the molecular mechanics Poisson–Boltzmann surface area (MM/PBSA) method. The van der Waals, electrostatic, polar solvation, and nonpolar solvation energy contributions were calculated from representative snapshots extracted from the equilibrated portion of the MD trajectory. The binding free energy was calculated according to the following equation:

ΔG_bind = G_complex − (G_protein + G_ligand)

where ΔG_bind represents the estimated binding free energy of HupA binding to AKT1, G_complex represents the free energy of the HupA–AKT1 complex, G_protein represents the free energy of unbound AKT1, and G_ligand represents the free energy of unbound HupA. Molecular dynamics simulations were performed in triplicate using independently generated initial velocities to ensure reproducibility.

### 2.4. Cell Culture and Experimental Design

H9c2 cells (ATCC, catalogue no. CRL-1446, Manassas, VA, USA) between passages 5 and 15 and authenticated by short tandem repeat (STR) profiling and confirmed to be mycoplasma-free were used in all experiments. H9c2 rat cardiomyoblast cells were cultured in high-glucose Dulbecco’s Modified Eagle Medium (DMEM) (Gibco, cat. no. C11995500BT, Grand Island, NY, USA) supplemented with 10% fetal bovine serum (FBS; VivaCell, Shanghai XP Biomed Ltd., Shanghai, China) and 1% penicillin–streptomycin (Pricella, Wuhan, China) at 37 °C in a humidified incubator containing 5% CO_2_. Cells were randomly assigned to the indicated treatment groups according to the specific experiment. Cells were seeded in 12-well plates at a density of 1.5 × 10^5^ cells per well, cultured overnight, and all treatments were initiated when the cells reached approximately 80% confluence.

For the oxidative stress model, cells were treated with 150 μM tert-butyl hydroperoxide (tBHP) (Macklin, Shanghai, China) for 3 h [23]. HupA (TargetMol, Shanghai, China) was used at a concentration of 10 μM and was added 0.5 h before tBHP exposure. For experiments involving AKT inhibition, cells were pretreated with 10 μM MK-2206 for 0.5 h before HupA and tBHP treatment [24]. The corresponding vehicle-treated cells received the same final concentration of the solvent without active compounds. The final solvent concentration was kept constant among the relevant treatment groups. Both HupA and MK-2206 were dissolved in DMSO (Solarbio, Beijing, China), and the final DMSO concentration was 0.1% (*v*/*v*) in all treatment groups, including the corresponding vehicle controls.

For the main tBHP/HupA experiments, the following groups were included: (1) Control group; (2) HupA-alone group; (3) tBHP group; and (4) HupA + tBHP group. For experiments evaluating the role of AKT signaling, the following groups were included: (1) Control group; (2) HupA-alone group; (3) tBHP group; (4) HupA + tBHP group; (5) MK-2206-alone group; (6) MK-2206 + tBHP group; and (7) MK-2206 + HupA + tBHP group, with the groups applied in each individual assay stated in the corresponding figure legend. The HupA + MK-2206 combination without tBHP was not included in the experimental design. This group was excluded since our hypothesis focuses on MK-2206 rescue of HupA-mediated protection under TBHP injury, and this basal combination group was unnecessary to test the research hypothesis. Therefore, the MK-2206-related experiments comprised seven treatment groups rather than the eight groups of a complete 2 × 2 × 2 factorial design.

### 2.5. Cell Viability Assay

Cell viability was assessed using the Cell Counting Kit-8 (CCK-8, cat. no. CK04-05; Dojindo Laboratories, Kumamoto, Japan) following the manufacturer’s instructions. Briefly, H9c2 cells were seeded in 96-well plates at a density of 8 × 10^3^ cells per well and subjected to the indicated treatments. Subsequently, 10 μL of CCK-8 solution was added to each well. After a 2 h incubation, the absorbance at 450 nm was detected with a microplate reader (Molecular Devices, San Jose, CA, USA).

### 2.6. Detection of Intracellular Reactive Oxygen Species

Intracellular reactive oxygen species (ROS) levels were evaluated using the 2′,7′-dichlorodihydrofluorescein diacetate (H2DCFDA) fluorescent probe (TargetMol, Shanghai, China). Following the indicated treatments, H9c2 cells were incubated with H2DCFDA staining solution at a final concentration of 5 µM for 60 min at 37 °C in the dark. After incubation, the cells were washed three times with PBS to remove excess extracellular probe and immediately imaged using a fluorescence microscope (YHF40; Shanghai Yuehe Biotechnology Co., Ltd., Shanghai, China). Images were acquired using identical acquisition parameters for all experimental groups (excitation 488 nm; emission 500–550 nm; exposure 35 ms; gain 500). For each biological replicate, five randomly selected fields were imaged from each well. Fluorescence intensity was quantified using ImageJ software (v1.53; https://imagej.net/ij/index.html; accessed on 21 June 2026), and the mean fluorescence intensity of the analyzed fields was calculated for each biological replicate before statistical analysis. Image quantification was performed in a blinded manner, and background fluorescence was subtracted using the region-of-interest (ROI) tool in ImageJ by selecting cell-free regions of each field prior to counting. The groups applied in each individual assay are stated in the corresponding figure legend.

### 2.7. Detection of Mitochondrial ROS

Mitochondrial superoxide production was assessed using the MitoSOX™ Red mitochondrial superoxide indicator (TargetMol, Shanghai, China). Following the indicated treatments, H9c2 cells were incubated with MitoSOX Red at a final concentration of 10 µM for 15 min at 37 °C in the dark. After staining, the cells were washed three times with PBS to remove excess probe and immediately subjected to fluorescence imaging using a fluorescence microscope (YHF40; Shanghai Yuehe Biotechnology Co., Ltd., Shanghai, China) equipped. Images were acquired under identical acquisition settings for all experimental groups using an excitation of 510 nm, an emission range of 570–620 nm, an exposure time of 179 ms, and a gain of 500. MitoSOX Red fluorescence intensity was quantified using ImageJ software (v1.53; https://imagej.net/ij/index.html; accessed on 21 June 2026). For each biological replicate, at least 3 non-overlapping imaged fields were captured and averaged to yield a single value for statistical analysis. The groups applied in each individual assay are stated in the corresponding figure legend.

### 2.8. Determination of Mitochondrial Membrane Potential

Mitochondrial membrane potential (ΔΨm) was assessed using an enhanced mitochondrial membrane potential assay kit with JC-1 (C2003S; Beyotime Biotechnology, Shanghai, China) according to the manufacturer’s instructions. Following the indicated treatments, H9c2 cells were incubated with 0.5 mL of JC-1 working solution per well at 37 °C for 20 min. After incubation, the staining solution was removed, and the cells were washed twice with JC-1 staining buffer. Subsequently, 1 mL of cell culture medium was added to each well, and the cells were immediately observed using a fluorescence microscope (YHF40; Shanghai Yuehe Biotechnology Co., Ltd., Shanghai, China). JC-1 monomers were detected using green fluorescence (excitation 488 nm; emission 500–550 nm; exposure 53 ms; gain 500), whereas JC-1 aggregates were detected using red fluorescence (excitation 514 nm; emission 580–650 nm; exposure 60 ms; gain 500). The red-to-green fluorescence intensity ratio was quantified using Image J software (v1.53; https://imagej.net/ij/index.html; accessed on 21 June 2026). For each biological replicate, at least 3 non-overlapping imaged fields were captured and averaged to yield a single value for statistical analysis. The groups applied in each individual assay are stated in the corresponding figure legend.

### 2.9. Hoechst 33342/PI Double Fluorescence Staining

Cell death was evaluated using Hoechst 33342/propidium iodide (PI) double fluorescence staining (TargetMol, Shanghai, China). Following the indicated treatments, H9c2 cells were stained with Hoechst 33342 (10 μg/mL) and PI (10 μg/mL) at 37 °C in the dark for 15 min according to the manufacturer’s instructions. Images were acquired using an inverted fluorescence microscope (YHF40; Shanghai Yuehe Biotechnology Co., Ltd., Shanghai, China). Images for Hoechst 33342 were acquired at an excitation of 405 nm with emission collected at 420–480 nm (exposure 61 ms, gain 500), and PI at an excitation of 535 nm with emission collected at 600–650 nm (exposure 61 ms, gain 500). Five non-overlapping fields were randomly selected from each well. Hoechst-positive and PI-positive cells were quantified using ImageJ (v1.53; https://imagej.net/ij/index.html; accessed on 21 June 2026), and the percentage of PI-positive cells was calculated as the number of PI-positive cells relative to the total number of Hoechst-positive cells. Image quantification was performed in a blinded manner, and background fluorescence was subtracted using the region-of-interest (ROI) tool in ImageJ by selecting cell-free regions of each field prior to counting. For each biological replicate, at least 3 non-overlapping imaged fields were captured and averaged to yield a single value for statistical analysis. The groups applied in each individual assay are stated in the corresponding figure legend.

### 2.10. Determination of Oxidative Stress Biomarkers

MDA content was determined via the thiobarbituric acid (TBA) colorimetric method, in which MDA reacts with TBA under high-temperature and acidic conditions to form a stable red adduct for colorimetric detection. SOD activity was measured using the xanthine oxidase assay, based on the inhibitory effect of SOD on the superoxide anion-mediated color reaction. GSH content was quantified by DTNB colorimetry, in which GSH reacts with DTNB to generate yellow chromogenic products for quantitative analysis. The intracellular levels of malondialdehyde (MDA, cat. no. A003-1-2), superoxide dismutase (SOD, cat. no. A001-3-2), and reduced glutathione (GSH, cat. no. A006-1-1) were measured using commercial assay kits following the manufacturers’ protocols (Nanjing Jiancheng Bioengineering Institute, Nanjing, China). Absorbance was determined using a microplate reader, and the results were normalized to the protein concentration. For each biological replicate, at least three technical replicate wells were measured and averaged to yield a single value for statistical analysis. The groups applied in each individual assay are stated in the corresponding figure legend.

### 2.11. Western Blot Analysis

The culture supernatants and corresponding cell pellets were collected following the indicated treatments. Total cellular proteins were extracted using RIPA lysis buffer (Thermo Fisher Scientific, Waltham, MA, USA) containing protease and phosphatase inhibitors (Thermo Fisher Scientific, Waltham, MA, USA). Protein concentrations were determined using a BCA protein assay kit (Pierce, Rockford, IL, USA) according to the manufacturer’s instructions. For extracellular HMGB1 analysis, the conditioned medium was collected in DMEM containing 10% FBS. Briefly, 300 μL of culture supernatant from each sample was centrifuged at 1000× *g* for 5 min to remove cell debris, and 20 μL of the resulting supernatant was loaded for Western blot analysis. The HMGB1 band intensity in culture supernatant was normalized to the total cellular protein content measured from the matching cell pellet of the same well. This normalization strategy accounts for treatment-induced variations in cell number and cell viability. The corresponding cell pellets were lysed with RIPA buffer, and the total cellular protein content was determined by BCA assay. For intracellular protein analysis, equal amounts of total cellular protein (20 μg per lane) were separated by SDS-PAGE on 10% polyacrylamide gels and electrophoretically transferred onto PVDF membranes (Merck Millipore, Burlington, MA, USA) at 110 V for 90 min. The membranes were blocked with 5% non-fat milk in TBST for 1 h at room temperature and then incubated overnight at 4 °C with primary antibodies against Akt1 (C73H10) Rabbit Monoclonal Antibody (#2938, 1:1000), Phospho-Akt1 (Ser473) (D7F10) Rabbit Monoclonal Antibody (#9018, 1:1000), Mcl-1 (D35A5) Rabbit Monoclonal Antibody (#5453, 1:1000), HMGB1 (D3E5) Rabbit Monoclonal Antibody (#6893, 1:1000), and GAPDH (D16H11) Rabbit Monoclonal Antibody (#5174, 1:1000) (Cell Signaling Technology, Danvers, MA, USA). After washing with TBST, the membranes were incubated with the corresponding HRP-conjugated anti-rabbit IgG secondary antibody (#7074; Cell Signaling Technology, Danvers, MA, USA; 1:2000) for 1 h at room temperature. Protein bands were visualized using an enhanced chemiluminescence (ECL) detection system and quantified using ImageJ software (v1.53). Intracellular protein expression levels were normalized to GAPDH, whereas extracellular HMGB1 levels were normalized to the total cellular protein content of the corresponding cell pellet. The *p*-AKT1 level was additionally expressed relative to total AKT1 where applicable. All Western blot experiments were independently performed using three biological replicates.

### 2.12. CETSA

Cellular thermal shift assay (CETSA) was performed to assess the thermal stability and cellular target engagement of AKT1 following HupA treatment. Briefly, H9c2 cardiomyoblasts were treated with HupA (10 μM) or the corresponding vehicle control for 3 h. A total of 5 × 10^5^ cells per experimental condition were harvested, washed with ice-cold phosphate-buffered saline (PBS), and resuspended in PBS at an equal cell density. Equal aliquots of the cell suspension were then subjected to a temperature gradient (37 °C, 41.6 °C, 46.2 °C, 50.8 °C, 55.4 °C, and 60 °C) for 3 min using a constant-temperature metal bath (Hangzhou Shenhua Technology Co., Ltd., Hangzhou, China), while the non-heated samples were maintained at 37 °C and served as the thermal control. Immediately after heating, all samples were cooled on ice for 5 min.

The cells were subsequently lysed using RIPA containing protease/phosphatase inhibitors and incubated on ice for 10 min. The lysates were centrifuged at 12,000 *g* for 15 min at 4 °C to remove insoluble and aggregated proteins. AKT1 protein levels were detected using the corresponding antibody, and the signal intensity at each temperature was quantified using ImageJ software.

For each temperature point, the relative soluble AKT1 level was normalized to the corresponding signal obtained at the lowest temperature/control condition. Thermal stability curves were generated by plotting the normalized soluble protein fraction against temperature. The melting curves from each biological replicate were fitted individually using a four-parameter logistic model with variable slope in GraphPad Prism (version 10.1.2; GraphPad Software, San Diego, CA, USA), and the apparent melting temperature (Tm) was defined as the temperature at which the normalized soluble AKT1 signal reached 50% of the fitted plateau. CETSA experiments were independently performed in 3 biological replicates.

### 2.13. LDH Releasing Assay

Lactate dehydrogenase (LDH) release was detected to assess cardiomyoblast injury using the Cytotoxicity Detection Kit PLUS (cat.no.:4744926001, LDH, Roche Diagnostics, Mannheim, Germany) according to the manufacturer’s protocol. Briefly, H9c2 cardiomyoblasts were pretreated with serial concentrations of HupA before t-BHP-induced oxidative stress. After intervention, an aliquot of cell culture supernatant was transferred into a new 96-well plate and mixed with the LDH reaction mixture. The plate was incubated at room temperature in the dark. Absorbance was measured at 490 nm using a microplate reader. Cells fully lysed with lysis buffer were set as the maximum LDH-release control, whereas untreated cells served as the basal control. The relative LDH release percentage was calculated to reflect the extent of cellular damage. For each biological replicate, at least three technical replicate wells were measured and averaged to yield a single value for statistical analysis. The groups applied in each individual assay are stated in the corresponding figure legend.

### 2.14. Statistical Analysis

Independent biological replicates were defined as cells cultured and treated in separate culture vessels on different days, and the exact number of biological replicates (n) is reported for each experiment in the corresponding figure legend. For fluorescence imaging experiments, at least 3 non-overlapping fields were randomly selected and imaged from each well, and field-level measurements were averaged within each biological replicate before statistical analysis. Data were presented as mean ± standard deviation (SD). Statistical analyses were performed using GraphPad Prism 10.1.2 and IBM SPSS Statistics 26.0 (IBM Corp., Armonk, NY, USA). For the seven-group experiments, global differences were assessed by one-way ANOVA. Pre-planned two-way factorial ANOVA was used to analyze tBHP × HupA, tBHP × MK-2206, and HupA × MK-2206 interactions, and partial η^2^ was reported as the effect size. The seven treatment groups were analyzed as two separate 2 × 2 factorial designs sharing common control groups, together with a mechanistic confirmation pair. The HupA × tBHP interaction (Ctrl, HupA, tBHP, tBHP + HupA) and the MK2206 × tBHP interaction (Ctrl, MK2206, tBHP, tBHP + MK2206) were each assessed by two-way factorial ANOVA with Tukey’s post hoc test; the effect of MK-2206 on HupA-mediated protection was tested by comparing tBHP + HupA with tBHP + HupA + MK2206 using an unpaired two-tailed Student’s *t*-test. For experiments involving a single experimental factor, one-way ANOVA followed by Tukey’s multiple-comparisons test was used. For comparisons between two groups, an unpaired two-tailed Student’s *t*-test was used. A significance threshold of *p* < 0.05 was applied. Exact F values, *p* values, and partial η^2^ effect sizes are reported in the Results section.

## 3. Results

### 3.1. Identification of Potential Targets and Enrichment Analysis of Huperzine A Against Cardiac Injury

**Figure 1:**
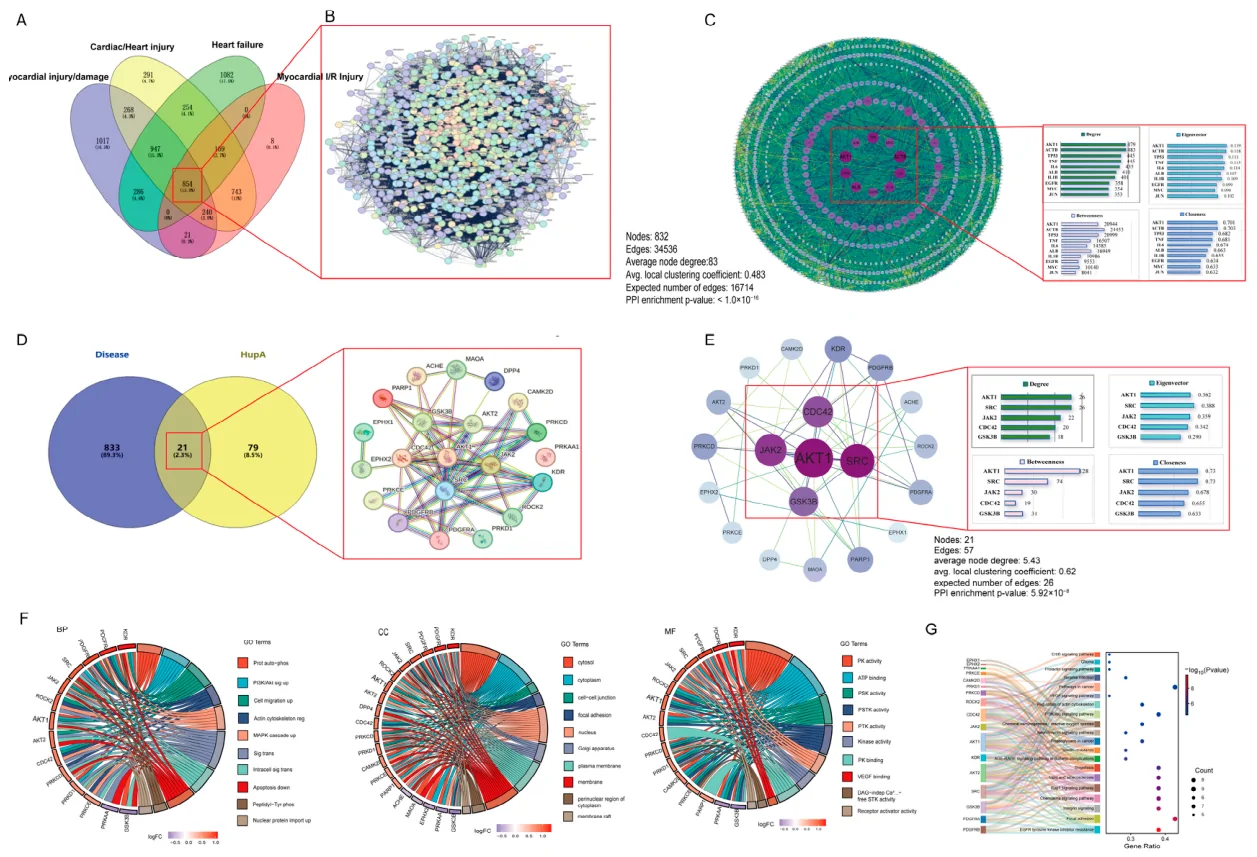
Network pharmacology analysis of Huperzine A against cardiac injury. (**A**) Venn diagram showing the overlap among disease-related targets associated with cardiac/heart injury, heart failure, myocardial injury/damage, and myocardial ischemia–reperfusion injury (MIRI). (**B**) Protein–protein interaction (PPI) network of the integrated cardiac injury-related targets. (**C**) Topological analysis of the cardiac injury-related PPI network based on Degree, Betweenness Centrality, Closeness Centrality, and Eigenvector Centrality. (**D**) Compound–target interaction network of Huperzine A and the overlapping therapeutic targets. (**E**) PPI network and hub gene analysis of the overlapping targets between Huperzine A and cardiac injury. (**F**) GO enrichment analysis. (**G**) KEGG pathway enrichment analysis.

To systematically explore the therapeutic mechanism of Huperzine A (HupA) against cardiac injury, disease-associated targets were collected from the CTD, GeneCards, and OMIM databases using the keywords “cardiac/heart injury”, “heart failure”, “myocardial injury/damage”, and “myocardial ischemia–reperfusion injury (MIRI)”. After removing duplicates, 6180 cardiac injury-related genes were obtained (Table 1). Among the targets associated with the four cardiac injury-related terms, 854 common targets were identified by Venn diagram analysis (Figure 1A), representing the shared targets among “cardiac/heart injury,” “heart failure,” “myocardial injury/damage,” and “myocardial ischemia–reperfusion injury (MIRI)” (Table S1). The PPI network constructed using the STRING database revealed extensive interactions among 854 genes (Figure 1B). Topological analysis based on Degree, Betweenness Centrality, Closeness Centrality, and Eigenvector Centrality identified several hub genes (Figure 1C). These 854 common targets were subsequently used for further intersection with HupA-related targets to identify potential therapeutic targets.

As shown in Figure 1D, a total of 21 common targets were identified between HupA-related targets and the 854 shared cardiac injury-related targets (Table S2). The PPI network constructed using the STRING database revealed extensive interactions among 21 genes (Figure 1D). Topological analysis based on Degree, Betweenness Centrality, Closeness Centrality, and Eigenvector Centrality identified several hub genes, including AKT1, SRC, and GSK3β (Figure 1E).

GO enrichment analysis demonstrated that these targets were mainly involved in the regulation of apoptotic signaling and protein phosphorylation (Figure 1F, Tables S3–S5). KEGG pathway analysis further indicated significant enrichment in the PI3K/AKT signaling pathway, apoptosis, MAPK signaling pathway, FoxO signaling pathway, and pathways related to cellular responses to oxidative stress (Figure 1G, Table S6). These findings suggested that HupA may alleviate myocardial injury primarily through modulation of oxidative stress- and survival-related signaling pathways. Among the identified hub targets, AKT1 was prioritized as a candidate target for further investigation based on the subsequent computational and experimental evidence.

### 3.2. Molecular Docking and Molecular Dynamics Simulations Predicted a Potential Interaction Between HupA and AKT1

**Figure 2:**
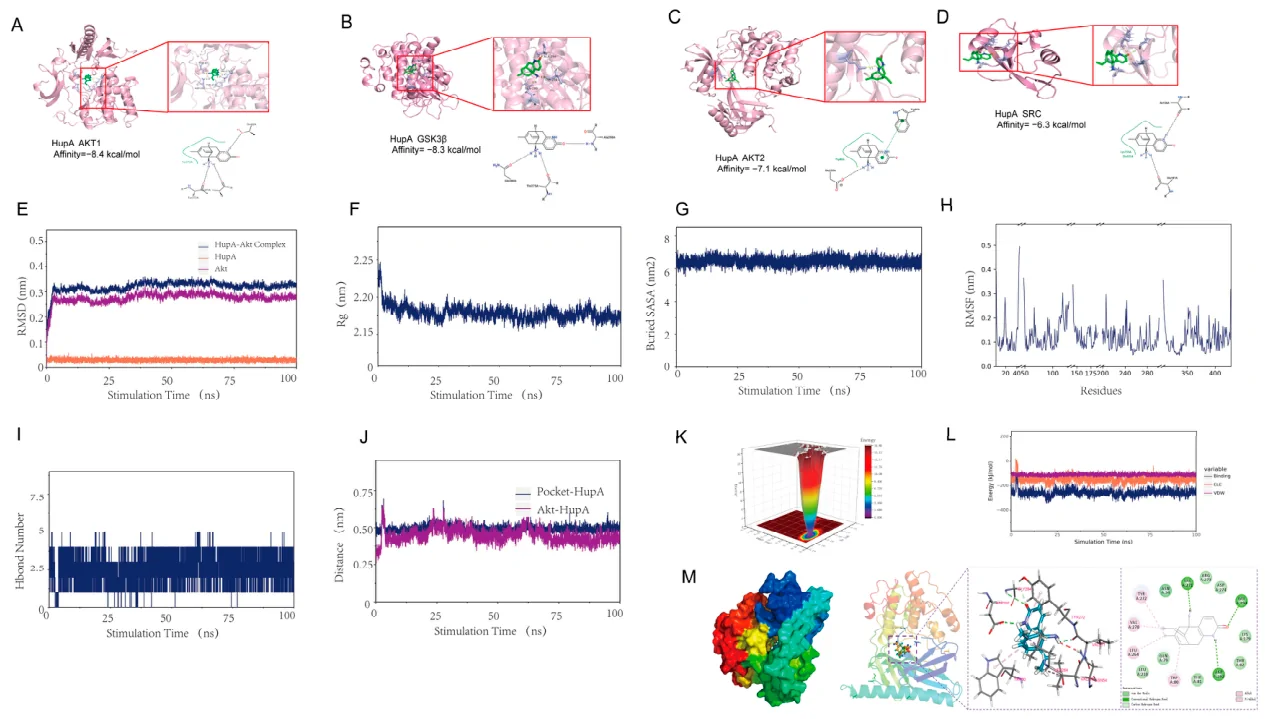
Molecular docking and molecular dynamics simulations of HupA with AKT1. (**A**–**D**) Docking conformations of HupA with AKT1, GSK3β, AKT2, and SRC. Corresponding binding affinities and two-dimensional interaction maps are shown. (**E**) Root-mean-square deviation (RMSD) trajectories of the HupA–AKT1 complex, free AKT1, and free HupA. (**F**) Radius of gyration (Rg) analysis. (**G**) Solvent-accessible surface area (SASA) analysis. (**H**) Root-mean-square fluctuation (RMSF) profile of AKT1 residues. (**I**) Number of hydrogen bonds formed between HupA and AKT1 during simulation. (**J**) Distance between HupA and the AKT1 binding pocket center. (**K**) Free-energy landscape of the HupA–AKT1 complex. (**L**) MM/PBSA binding free-energy decomposition. (**M**) Surface representation of the AKT1 binding pocket and key residue interactions with HupA.

To further explore the potential interaction between HupA and the hub proteins identified by network pharmacology, molecular docking was performed against AKT1, AKT2, GSK3β and SRC. The resulting binding affinities were −8.4, −7.1, −8.3 and −6.3 kcal/mol for AKT1, AKT2, GSK3β and SRC, respectively (Figure 2A–D). Therefore, AKT1 was selected for subsequent molecular dynamics simulations because it exhibited a well-defined docking pose with favorable contacts within the binding site and, more importantly, represents a central hub with established cardioprotective relevance in the network pharmacology analysis. Before these docking poses were interpreted, the reliability of the docking protocol was validated by redocking. The co-crystallized ligand XOO was extracted from the AKT1 crystal structure (PDB ID: 8UW7, 2.00 Å resolution) and used as the reference. Protein preprocessing (hydrogen addition, charge assignment, and protonation at pH 7.4), ligand preparation, grid box definition, and docking parameters (scoring function, exhaustiveness, and protein flexibility settings) were kept identical to those used for the candidate compounds in this study. XOO was then redocked into the binding pocket, and the top-ranked pose was selected for validation. After superimposing the protein Cα atoms, the heavy-atom RMSD between the redocked pose and the native crystal conformation of XOO was calculated in PyMOL. The obtained heavy-atom RMSD was 1.60 Å (Figure S2), below the 2.0 Å cutoff commonly used to assess whether a docking protocol can recapitulate the experimentally determined binding mode, confirming the reliability of the docking workflow used in this study.

The stability of the AKT1–HupA complex was subsequently evaluated by 100 ns molecular dynamics simulation (Figure 2 and Figure S2). The complex RMSD increased during the first 5 ns of equilibration and then reached a stable plateau. Over the equilibrated phase (50–100 ns), the mean RMSD was 3.12 ± 0.22 Å and no significant drift, indicating that the complex remained structurally stable (Figure 2E). Consistently, the radius of gyration (Rg) of the complex remained essentially constant throughout the 100 ns simulation, with a mean value of 2.19 ± 0.01 nm (21.9 ± 0.12 Å) and a relative fluctuation of only 0.56% (0.52% over the equilibrated phase), indicating that the complex retained a compact, well-folded conformation without unfolding or significant structural expansion. The solvent-accessible surface area (SASA) of the complex remained stable throughout the 100 ns simulation, with a mean value of 200.0 ± 4.4 nm^2^ (2.00 ± 0.04 × 10^4^Å^2^) over the entire trajectory and a relative fluctuation of only 2.18%, consistent with a well-packed and solvent-stable complex (Figure 2F,G). Per-residue root-mean-square fluctuation (RMSF) analysis showed that the protein remained globally rigid throughout the simulation, with a mean RMSF of 1.33 ± 0.84 Å and a median of 1.09 Å across all residues. About 65% of residues exhibited RMSF below the average. Elevated fluctuations were confined to the flexible terminal residues (up to 3.2 Å at the C-terminus) and several solvent-exposed loops (residues 44–49, 123–129, and 307–308, with local maxima of 4–6 Å), whereas the core structural elements remained stable (Figure 2H). Hydrogen-bond analysis showed that HupA formed persistent yet dynamically fluctuating hydrogen bonds with AKT1, with a time-averaged count of 2.32 ± 0.86. At least one hydrogen bond was present in 98.8% of the trajectory frames, with up to six simultaneous H-bonds. These results indicate that after equilibration, HupA approached and remained accommodated within the binding-site region throughout the simulations (Figure 2I,J). The free-energy landscape demonstrated a dominant low-energy conformational state (Figure 2K), while MM/PBSA analysis revealed a favorable binding free energy (ΔGbind = −59.60 ± 9.86 kcal/mol), with van der Waals interactions serving as the major driving force for complex formation (Table 2 and Figure 2L). As shown in Figure 2M, multiple pocket residues of Akt engage in intermolecular contacts with HupA. Hydrogen bonds were formed between HupA and the residues Val271, Gly294, and Asp292, whereas hydrophobic interactions of the alkyl and π-alkyl types were observed with Tyr272, Val270, Leu264, and Trp80. Van der Waals contacts were predominantly mediated by Leu210, Lys179, and Arg273, among others. Collectively, the combination of hydrogen bonding and hydrophobic interactions provides a structural hypothesis for the interaction between HupA and AKT1.

### 3.3. Huperzine A Protects H9c2 Cardiomyoblasts Against tBHP-Induced Cytotoxicity

**Figure 3:**
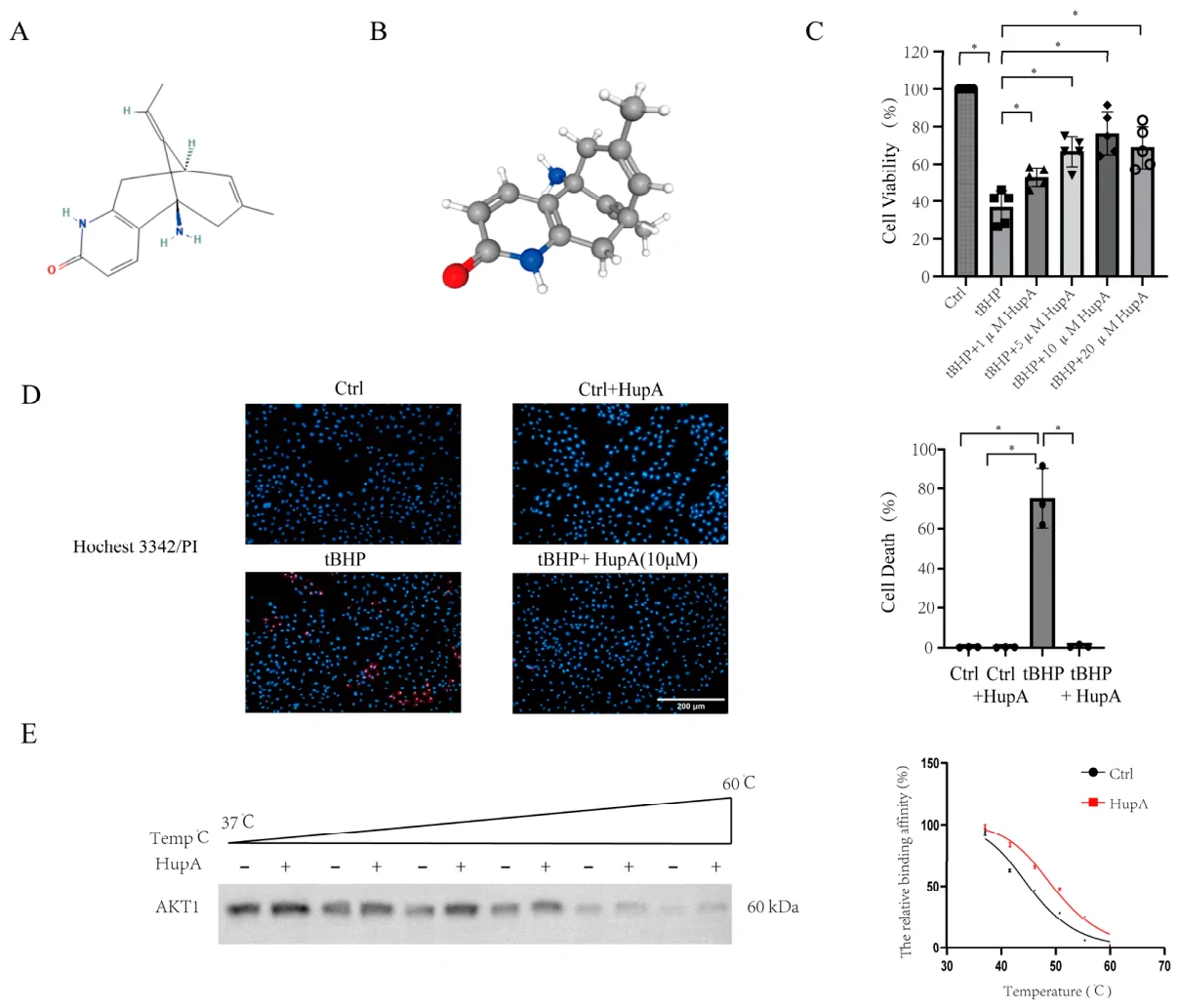
Huperzine A protects H9c2 cardiomyoblasts against tBHP-induced cytotoxicity. (**A**) Two-dimensional chemical structure of Huperzine A (HupA). (**B**) Three-dimensional molecular structure of Huperzine A. (**C**) Effects of different concentrations of HupA on the viability of H9c2 cardiomyoblasts exposed to tert-butyl hydroperoxide (tBHP). Cell viability was assessed by CCK-8 after pretreatment with HupA (1–20 μM) followed by tBHP exposure. HupA significantly improved cell viability in a concentration-dependent manner, with 10 μM selected for subsequent experiments. (**D**) Representative fluorescence images of Hoechst 33342/propidium iodide (PI) staining and quantitative analysis of PI-positive cells. Hoechst 33342 (blue) stains all nuclei, whereas PI (red) labels dead cells with compromised membrane integrity. HupA (10 μM) markedly reduced tBHP-induced cell death in H9c2 cardiomyoblasts. (**E**) Cellular thermal shift assay (CETSA) assessing cellular target engagement of AKT1 by HupA. Left panel: Representative immunoblot bands of AKT1 protein after gradient temperature treatment with or without 10 μM HupA. Right panel: Quantitative fitting curves showing the normalized soluble fraction of AKT1 along the temperature gradient, fitted separately for each biological replicate (n = 3). HupA treatment shifted the melting curve of AKT1 toward higher temperature, indicating that HupA increases the thermal stability of AKT1 in cells. Each dot represents an independent biological replicate. Data are presented as the mean ± SD. For cell viability assay, statistical significance was determined by one-way ANOVA followed by Tukey’s multiple-comparisons test (n = 5). For Hoechst 33342/PI staining (n = 3), statistical significance was determined by two-way ANOVA (tBHP × HupA): tBHP main effect F (1,8) = 75.79, *p* = 2.4 × 10^−5^, partial η^2^ = 0.905; HupA main effect F (1,8) = 73.62, *p* = 2.6 × 10^−5^, partial η^2^ = 0.902; HupA × tBHP interaction F (1,8) = 73.46, *p* = 2.7 × 10^−5^, partial η^2^ = 0.902. For CETSA (n = 3), the thermal shift (ΔTm) was statistically significant (paired two-tailed Student’s *t*-test, *p* = 0.002). * *p* < 0.05.

The chemical structure of HupA is shown in Figure 3A,B. To determine an appropriate experimental concentration, H9c2 cells were pretreated with increasing concentrations of HupA. To determine an appropriate concentration of HupA for subsequent experiments, a concentration-response analysis was first performed using HupA at 1, 5, 10, and 20 μM. As shown in Figure S1A, HupA alone did not significantly affect basal H9c2 cell viability at concentrations up to 10 μM, whereas a modest reduction in viability was observed at 20 μM. We next examined the protective effects of these concentrations against tBHP-induced cytotoxicity. As shown in Figure 3C, tBHP markedly reduced cell viability, whereas HupA pretreatment progressively improved cell viability across the tested concentration range. Among the concentrations that maintained acceptable basal cell viability, 10 μM provided a robust protective effect against tBHP-induced injury and was therefore selected for subsequent mechanistic experiments. Thus, 10 μM HupA was used throughout the subsequent experiments.

Hoechst 33342/PI staining further confirmed the cytoprotective effect of HupA. As shown in Figure 3D, tBHP exposure markedly increased PI-positive cells from 0.31% ± 0.15% (control) to 75.29% ± 15.02%, and HupA pretreatment markedly reduced this proportion to 0.86% ± 0.62% (HupA alone: 0.27% ± 0.10%). Because PI fluorescence indicates loss of plasma-membrane integrity, these changes reflect pronounced tBHP-induced cell death and its attenuation by HupA. Two-way ANOVA (n = 3) revealed significant main effects of tBHP (F(1,8) = 75.79, *p* = 2.4 × 10^−5^, partial η^2^ = 0.905) and HupA (F(1,8) = 73.62, *p* = 2.6 × 10^−5^, partial η^2^ = 0.902) and a significant tBHP × HupA interaction (F(1,8) = 73.46, *p* = 2.7 × 10^−5^, partial η^2^ = 0.902), indicating that HupA effectively attenuated tBHP-induced cardiomyoblast injury.

We next performed a cellular thermal shift assay (CETSA) to investigate whether HupA engages AKT1 within intact cells. Gradient temperature heating induced progressive thermal denaturation of AKT1 in the control group. Notably, incubation with HupA shifted the AKT1 melting curve toward higher temperatures, indicating enhanced thermal stability of AKT1 (Figure 3E). Melting curves were fitted with a four-parameter nonlinear model for each biological replicate, yielding a mean fitted Tm of 45.11 ± 0.17 °C (95% CI: 44.68–45.53 °C) for the control group and 49.48 ± 0.17 °C (95% CI: 49.07–49.89 °C) for the HupA-treated group, corresponding to a mean thermal shift (ΔTm) of 4.37 ± 0.34 °C (range: 4.01–4.67 °C across individual replicates; individual fitted Tm values and 95% CIs are provided in Figure S3). All fits showed excellent goodness of fit (R^2^ = 0.97–0.98). These findings indicate that HupA engages with AKT1 and increases its thermal stability, supporting AKT1 as a potential molecular target of HupA and providing a potential molecular basis for the protective function of HupA in H9c2 cardiomyoblasts.

### 3.4. Huperzine A Attenuates Oxidative Stress and Mitochondrial ROS Generation

To evaluate whether the protective effect of HupA was associated with the attenuation of oxidative stress, intracellular oxidative stress markers were examined. Intracellular ROS and mitochondrial superoxide levels were assessed by DCFH-DA and MitoSOX Red staining, respectively, and MDA content, SOD activity, and GSH content were measured using commercial assay kits.

**Figure 4:**
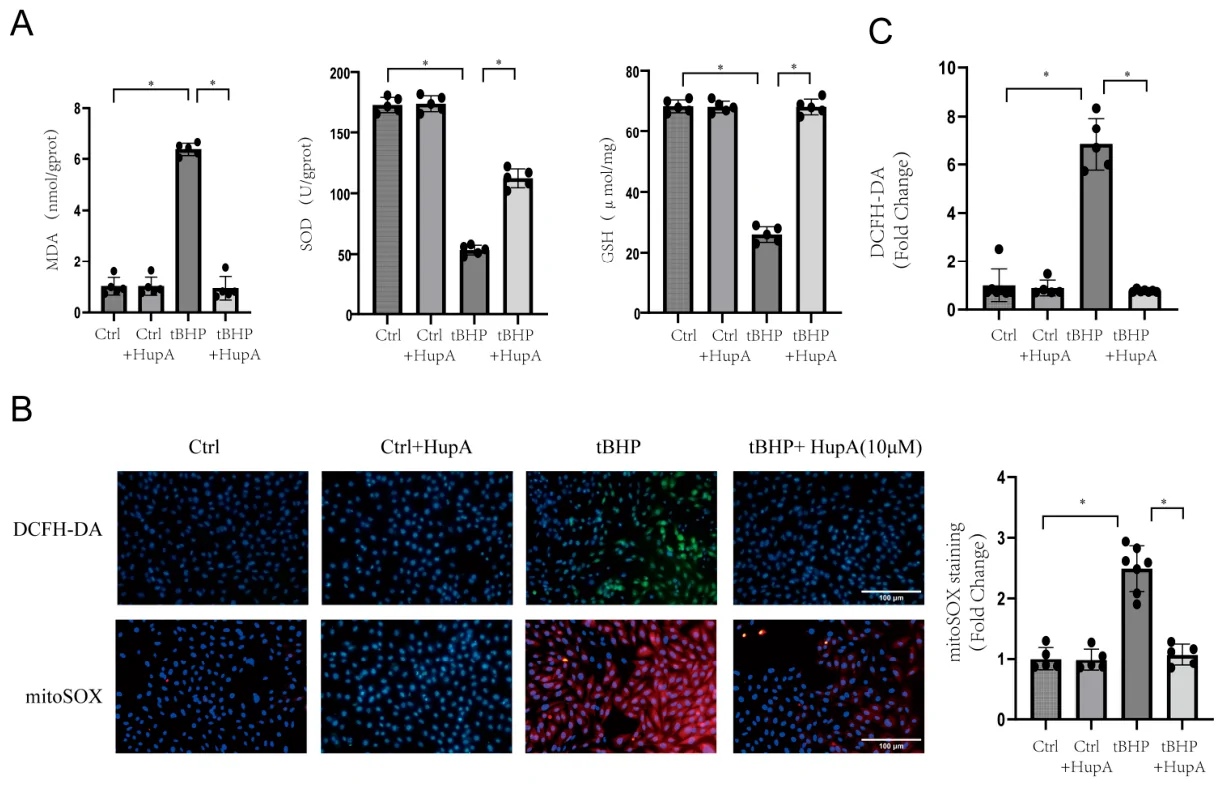
Huperzine A attenuates tBHP-induced oxidative stress and mitochondrial ROS generation in H9c2 cardiomyoblasts. (**A**) Effects of Huperzine A (HupA, 10 μM) on intracellular oxidative stress-related biomarkers following tert-butyl hydroperoxide (tBHP)-induced injury. Intracellular malondialdehyde (MDA) content, superoxide dismutase (SOD) activity, and glutathione (GSH) levels were determined after the indicated treatments. tBHP markedly increased lipid peroxidation while decreasing SOD activity and GSH content, whereas HupA pretreatment significantly reversed these alterations. (**B**) Representative fluorescence images of intracellular reactive oxygen species (ROS) detected by DCFH-DA staining (green) and mitochondrial superoxide detected by MitoSOX Red staining (red); nuclei were counterstained with DAPI (blue). Intracellular ROS was detected by DCFH-DA, and mitochondrial superoxide by MitoSOX Red. (**C**) Quantitative analysis of DCFH-DA and MitoSOX Red fluorescence intensities. Fluorescence signals were normalized to the control group and expressed as fold change. Each dot represents an independent biological replicate. * *p* < 0.05. Data are presented as the mean ± SD. For MDA (n = 5 per group): tBHP main effect F(1,16) = 271.01, *p* < 0.001, partial η^2^ = 0.944; HupA main effect F(1,16) = 287.30, *p* < 0.001, partial η^2^ = 0.947; HupA × tBHP interaction F(1,16) = 287.72, *p* < 0.001, partial η^2^ = 0.947. For GSH content (n = 5 per group): tBHP main effect F(1,16) = 424.00, *p* < 0.001, partial η^2^ = 0.964; HupA main effect F(1,16) = 416.04, *p* < 0.001, partial η^2^ = 0.963; HupA × tBHP interaction F(1,16) = 424.00, *p* < 0.001, partial η^2^ = 0.964. For SOD activity (n = 5 per group): tBHP main effect F(1,16) = 1050.27, *p* < 0.001, partial η^2^ = 0.985; HupA main effect F(1,16) = 114.65, *p* < 0.001, partial η^2^ = 0.878; HupA × tBHP interaction F(1,16) = 107.13, *p* < 0.001, partial η^2^ = 0.870. For DCFH-DA fluorescence intensity (n = 5 per group): tBHP main effect F(1,16) = 61.32, *p* < 0.001, partial η^2^ = 0.793; HupA main effect F(1,16) = 78.26, *p* < 0.001, partial η^2^ = 0.830; HupA × tBHP interaction F(1,16) = 77.48, *p* < 0.001, partial η^2^ = 0.829. For MitoSOX Red fluorescence intensity (n = 5 per group): tBHP main effect F(1,16) = 43.49, *p* < 0.001, partial η^2^ = 0.731; HupA main effect F(1,16) = 35.99, *p* < 0.001, partial η^2^ = 0.692; HupA × tBHP interaction F(1,16) = 34.40, *p* < 0.001, partial η^2^ = 0.683.

As shown in Figure 4A, tBHP treatment significantly increased MDA content (6.37 ± 0.24 vs. 1.02 ± 0.34 nmol/g prot) while markedly reducing GSH content (26.00 ± 2.55 vs. 68.40 ± 2.07 μmol/mg) and SOD activity (53.40 ± 3.97 vs. 173.20 ± 6.22 U/gprot) compared with the control group. HupA pretreatment largely reversed these changes: MDA (0.94 ± 0.46 nmol/gprot) and GSH (68.20 ± 2.59 μmol/mg) were restored to control levels, whereas SOD activity was only partially restored (112.40 ± 7.77 U/g prot) and remained significantly lower than control levels (*p* < 0.001), indicating partial recovery of intracellular antioxidant capacity. Two-way ANOVA (n = 5 per group) confirmed significant main effects of tBHP and HupA and a significant interaction for all three markers (MDA: F(1,16) = 271.01, 287.30, and 287.72; GSH: F(1,16) = 424.00, 416.04, and 424.00; SOD: F(1,16) = 1050.27, 114.65, and 107.13; all *p* < 0.001; partial η^2^ = 0.944/0.947/0.947, 0.964/0.963/0.964, and 0.985/0.878/0.870, respectively).

As shown in Figure 4B tBHP exposure markedly increased mitochondrial superoxide levels (2.41 ± 0.39 vs. 1.00 ± 0.19 in the control group), whereas HupA pretreatment reduced mitochondrial superoxide production to a level comparable to that of the control (1.07 ± 0.18; simple effect *p* = 0.567). Two-way ANOVA (HupA × tBHP, n = 5 per group) revealed significant main effects of tBHP (F(1,16) = 43.49, *p* < 0.001, partial η^2^ = 0.731) and HupA (F(1,16) = 35.99, *p* < 0.001, partial η^2^ = 0.692) and a significant interaction (F(1,16) = 34.40, *p* < 0.001, partial η^2^ = 0.683).

Similarly, intracellular ROS levels were markedly elevated after tBHP exposure (6.85 ± 1.08 vs. 1.10 ± 0.79 in the control group) and restored to control levels by HupA pretreatment (0.75 ± 0.03; simple effect *p* = 0.351) (Figure 4B,C). Two-way ANOVA revealed significant main effects of tBHP (F(1,16) = 61.32, *p* < 0.001, partial η^2^ = 0.793) and HupA (F(1,16) = 78.26, *p* < 0.001, partial η^2^ = 0.830) and a significant interaction (F(1,16) = 77.48, *p* < 0.001, partial η^2^ = 0.829).

Together, these results demonstrate that HupA alleviated tBHP-induced oxidative stress in H9c2 cardiomyoblasts by suppressing ROS accumulation and partially restoring the antioxidant defense system.

### 3.5. Huperzine A Preserves Mitochondrial Membrane Potential

**Figure 5:**
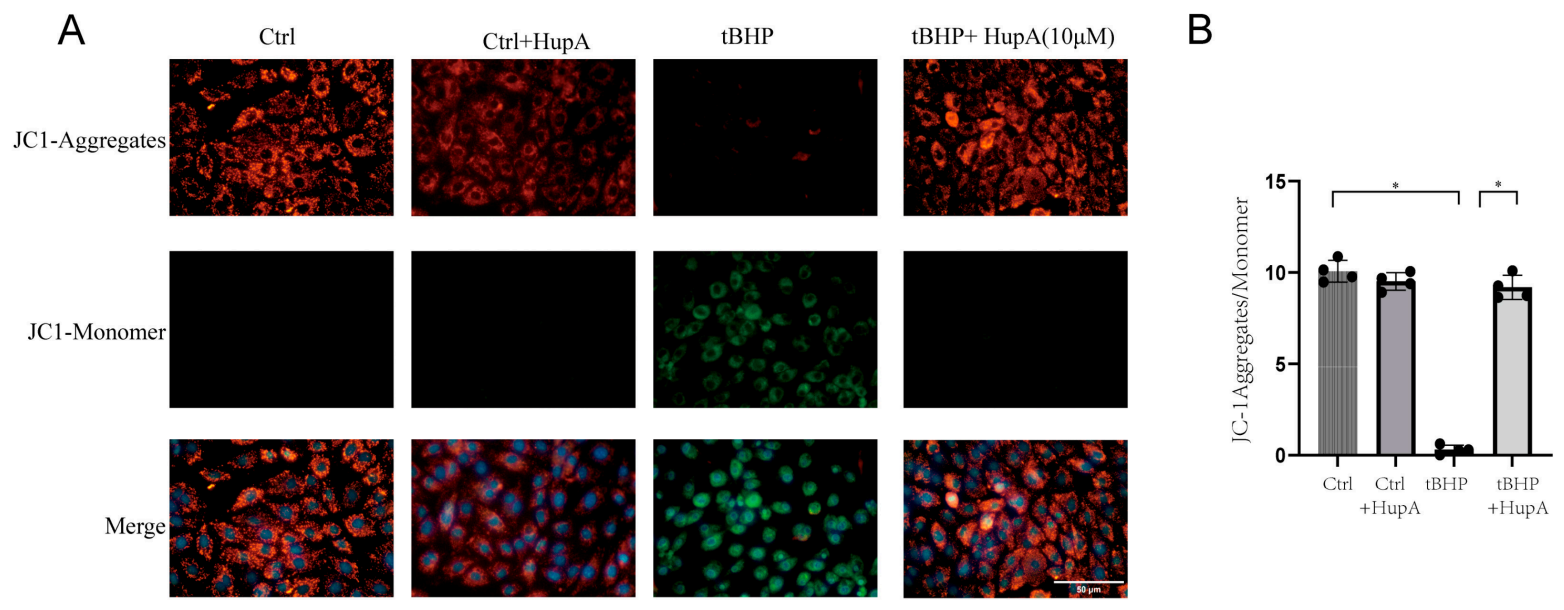
Huperzine A preserves mitochondrial membrane potential in tBHP-treated H9c2 cardiomyoblasts. (**A**) Representative fluorescence images of JC-1 staining in H9c2 cardiomyoblasts following the indicated treatments. Healthy mitochondria with intact mitochondrial membrane potential (ΔΨm) exhibited predominant JC-1 aggregate fluorescence (red), whereas depolarized mitochondria displayed increased JC-1 monomer fluorescence (green). Nuclei were counterstained with DAPI (blue). Treatment with tert-butyl hydroperoxide (tBHP) markedly induced mitochondrial membrane depolarization, as evidenced by decreased red fluorescence and increased green fluorescence, while Huperzine A (HupA, 10 μM) substantially restored mitochondrial membrane potential. (**B**) Quantitative analysis of the JC-1 aggregate/monomer fluorescence intensity ratio. Each dot represents an independent biological replicate. Data are presented as the mean ± SD. For the JC-1 aggregate/monomer fluorescence ratio (n = 4 per group): tBHP main effect F(1,12) = 410.58, *p* = 1.2 × 10^−10^, partial η^2^ = 0.972; HupA main effect F(1,12) = 286.49, *p* = 9.7 × 10^−10^, partial η^2^ = 0.960; HupA × tBHP interaction F(1,12) = 283.06, *p* = 1.0 × 10^−9^, partial η^2^ = 0.959. * *p* < 0.05.

Given the close association between excessive oxidative stress and mitochondrial dysfunction, mitochondrial membrane potential (ΔΨm) was assessed by JC-1 staining. As shown in Figure 5A, control cells predominantly exhibited red fluorescence corresponding to JC-1 aggregates, whereas tBHP exposure markedly increased green JC-1 monomer fluorescence, indicating mitochondrial membrane depolarization; HupA pretreatment substantially restored red fluorescence and reduced green fluorescence.

Quantitative analysis revealed that the JC-1 aggregate/monomer (red/green) ratio, a sensitive indicator of ΔΨm, was markedly decreased by tBHP exposure (0.30 ± 0.24 vs. 10.07 ± 0.60 in the control group) and effectively restored by HupA pretreatment (9.19 ± 0.66), which was not significantly different from control levels (simple effect *p* = 0.096), whereas HupA alone had no marked effect on the ratio (10.09 ± 0.50) (Figure 5B). Two-way ANOVA (HupA × tBHP, n = 4 per group) confirmed significant main effects of tBHP (F(1,12) = 410.58, *p* = 1.2 × 10^−10^, partial η^2^ = 0.972) and HupA (F(1,12) = 286.49, *p* = 9.7 × 10^−10^, partial η^2^ = 0.960) and a significant HupA × tBHP interaction (F(1,12) = 283.06, *p* = 1.0 × 10^−9^, partial η^2^ = 0.959), indicating that HupA protected against tBHP-induced ΔΨm collapse and preserved mitochondrial membrane integrity.

### 3.6. Huperzine A Modulates AKT-Associated Signaling in tBHP-Treated H9c2 Cardiomyoblasts

**Figure 6:**
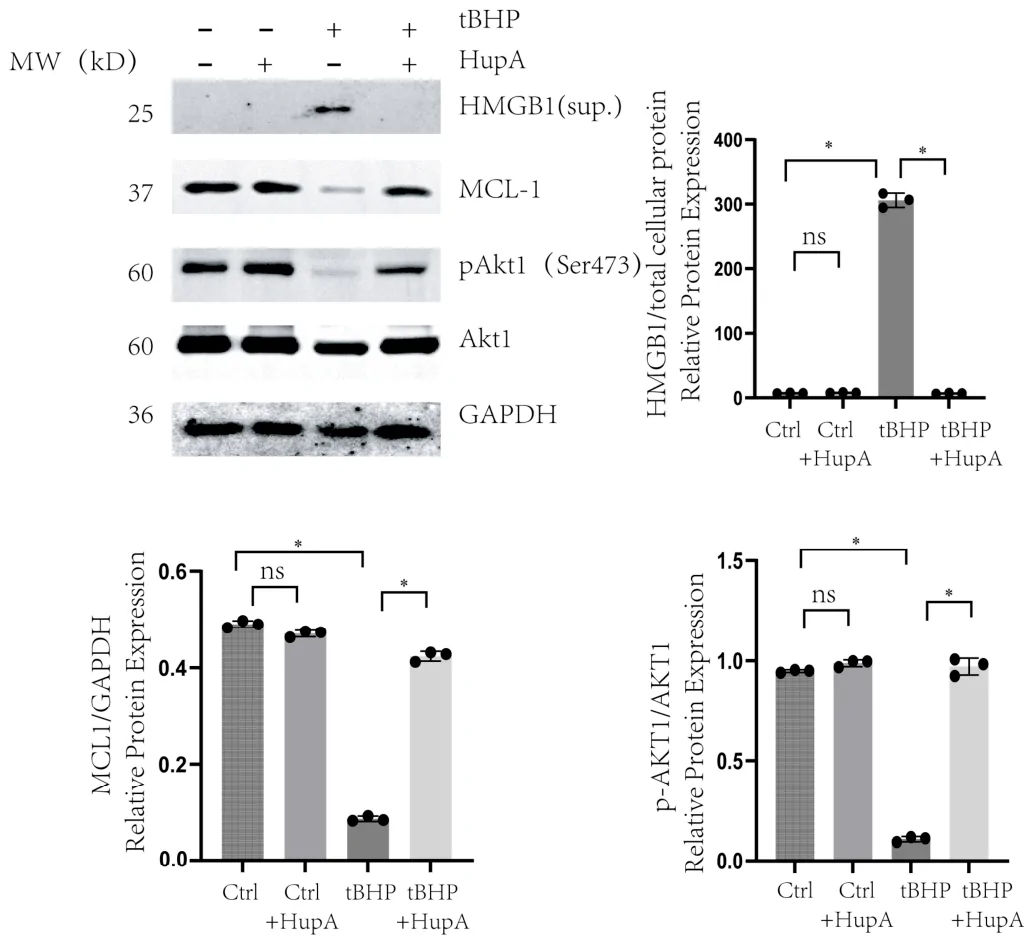
Huperzine A modulates AKT-associated signaling in tBHP-treated H9c2 cardiomyoblasts. Representative Western blot images showing the protein expression of extracellular HMGB1 (supernatant), MCL-1, phosphorylated AKT1 (Ser473), total AKT1, and GAPDH in H9c2 cardiomyoblasts following the indicated treatments. Quantitative analyses of HMGB1/total cellular protein, MCL-1/GAPDH, and *p*-AKT1/AKT1 protein expression are shown in the corresponding bar graphs. Exposure to tert-butyl hydroperoxide (tBHP) significantly increased HMGB1 release while decreasing MCL-1 expression and AKT1 phosphorylation. Pretreatment with Huperzine A (HupA, 10 μM) markedly suppressed HMGB1 release and restored MCL-1 expression and AKT1 phosphorylation. Data are presented as the mean ± SD from three independent experiments. Protein expression levels were normalized to GAPDH, whereas phosphorylated AKT1 was normalized to total AKT1. Data are presented as the mean ± SD from three independent biological replicates. Protein expression levels were normalized to GAPDH, phosphorylated AKT1 was normalized to total AKT1, and extracellular HMGB1 was normalized to total cellular protein. For extracellular HMGB1: tBHP main effect F(1,8) = 2145.75, *p* = 5.2 × 10^−11^, partial η^2^ = 0.996; HupA main effect F(1,8) = 2159.59, *p* = 5.1 × 10^−11^, partial η^2^ = 0.996; HupA × tBHP interaction F(1,8) = 2177.23, *p* = 4.9 × 10^−11^, partial η^2^ = 0.996. For MCL-1/GAPDH: tBHP main effect F(1,8) = 2606.75, *p* = 2.4 × 10^−11^, partial η^2^ = 0.997; HupA main effect F(1,8) = 1298.20, *p* = 3.9 × 10^−10^, partial η^2^ = 0.994; HupA × tBHP interaction F(1,8) = 1632.01, *p* = 1.6 × 10^−10^, partial η^2^ = 0.995. For *p*-AKT1/AKT1: tBHP main effect F(1,8) = 964.33, *p* = 1.3 × 10^−9^, partial η^2^ = 0.992; HupA main effect F(1,8) = 1069.12, *p* = 8.4 × 10^−10^, partial η^2^ = 0.993; HupA × tBHP interaction F(1,8) = 893.55, *p* = 1.7 × 10^−9^, partial η^2^ = 0.991. ns, not significant. * *p* < 0.05.

To investigate the molecular mechanism underlying the protective effects of HupA, the expression of AKT-associated signaling proteins was analyzed by Western blotting. As shown in Figure 6, tBHP exposure significantly reduced AKT1 phosphorylation and MCL-1 expression while markedly increasing extracellular HMGB1 release; HupA pretreatment restored AKT1 phosphorylation and MCL-1 protein levels and suppressed HMGB1 release into the culture supernatant.

Specifically, tBHP exposure markedly reduced the *p*-AKT1/AKT1 ratio (0.109 ± 0.013 vs. 0.949 ± 0.007 in the control group), which was fully restored by HupA pretreatment (0.972 ± 0.042; HupA alone: 0.988 ± 0.017). Consistently, MCL-1 protein levels normalized to GAPDH were markedly decreased by tBHP exposure (0.086 ± 0.006 vs. 0.490 ± 0.007 in the control group) and largely restored by HupA pretreatment (0.424 ± 0.010; HupA alone: 0.471 ± 0.007). Conversely, extracellular HMGB1 levels in the culture supernatant were markedly increased by tBHP exposure (305.96 ± 11.07 vs. 7.29 ± 0.58 in the control group) and were abolished by HupA pretreatment (6.81 ± 0.75; HupA alone: 7.90 ± 0.56). Together, these findings demonstrate that HupA activated AKT signaling in tBHP-challenged cells, as reflected by restored AKT1 phosphorylation and MCL-1 expression, and suppressed HMGB1 release, suggesting that the AKT1-associated signaling may contribute to the cardioprotective effects of HupA under oxidative stress.

### 3.7. AKT Inhibition Abolishes the Protective Effects of Huperzine A

**Figure 7:**
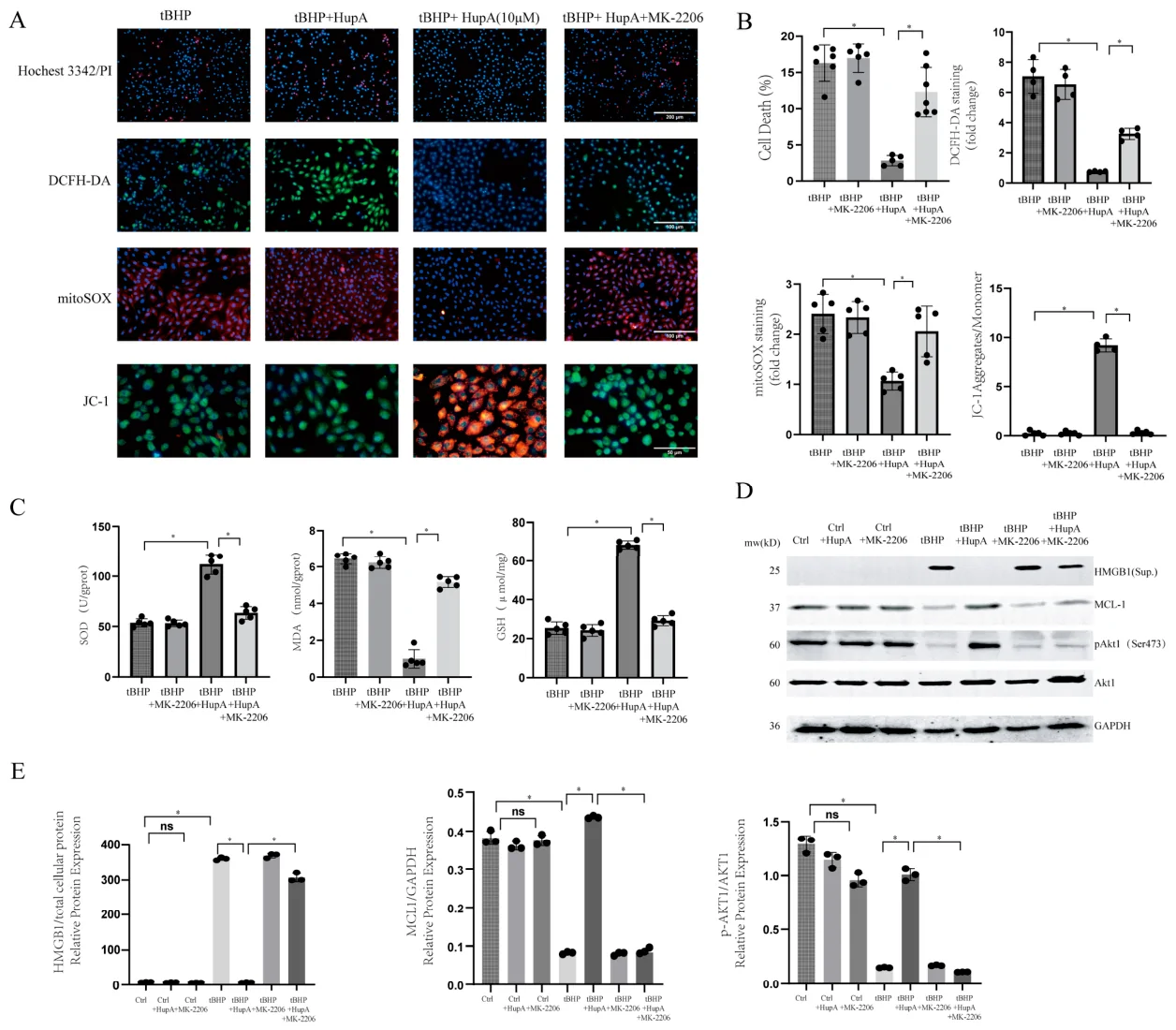
Inhibition of AKT1 signaling abolishes the protective effects of Huperzine A against tBHP-induced oxidative stress and mitochondrial dysfunction in H9c2 cardiomyoblasts. (**A**) Representative fluorescence images of Hoechst 33342/PI staining, DCFH-DA staining, MitoSOX Red staining, and JC-1 staining in H9c2 cardiomyoblasts following the indicated treatments. Cell death was assessed by Hoechst 33342/PI staining, intracellular reactive oxygen species (ROS) were detected using DCFH-DA, mitochondrial superoxide production was evaluated by MitoSOX Red staining, and mitochondrial membrane potential (ΔΨm) was determined by JC-1 staining. Pretreatment with Huperzine A (HupA, 10 μM) markedly reduced tBHP-induced cell death, intracellular ROS accumulation, mitochondrial ROS production, and mitochondrial membrane depolarization, whereas these protective effects were largely abolished by the AKT inhibitor MK-2206. (**B**) Quantitative analysis of PI-positive cell death (n = 6), DCFH-DA fluorescence intensity (n = 4), MitoSOX fluorescence intensity (n = 5), and the JC-1 aggregate/monomer fluorescence ratio (n = 4). Fluorescence intensities were normalized to the tBHP group and expressed as fold change where indicated. (**C**) Effects of AKT inhibition on oxidative stress-related biochemical parameters, including superoxide dismutase (SOD) activity, malondialdehyde (MDA) content, and glutathione (GSH) levels. MK-2206 significantly attenuated the HupA-mediated restoration of antioxidant capacity and promoted oxidative stress following tBHP exposure. (**D**) Western blot bands showing the protein expression of HMGB1 (supernatant), MCL-1, *p*-Akt1 (Ser473) and total Akt1 (**E**) Quantitative statistical analysis of relative protein levels of HMGB1/total cellular protein, MCL-1/GAPDH, and *p*-Akt1/Akt1 ratio (n = 3). Each dot represents an independent biological replicate. Data are presented as the mean ± SD. For Western blot, one-way ANOVA revealed significant overall differences across the seven experimental groups for all indicators (*p*-AKT1/AKT1: F = 330.588, *p* < 0.0001, η^2^ = 0.993; MCL1: F = 836.44, *p* < 0.0001, η^2^ = 0.997; HMGB1: F = 3295.2, *p* < 0.0001, η^2^ = 0.999). Pre-planned HupA × tBHP and MK-2206 × tBHP factorial designs were analyzed by two-way ANOVA followed by Tukey’s post hoc test; the tBHP + HupA versus tBHP + HupA + MK-2206 comparison was performed with an unpaired two-tailed Student’s *t*-test. F, *p*, partial η^2^, Cohen’s d are reported in Table S7. Summary of statistical analyses for *p*-AKT1/AKT1, MCL-1, and HMGB1 in H9c2 cells *p* < 0.0001; ns, not significant. * *p* < 0.05.

To further examine whether AKT1 activation contributes to the protective effects of HupA, H9c2 cells were treated with the selective AKT inhibitor MK-2206 (TargetMol, Shanghai, China) before HupA administration. As shown in Figure 7A,B, MK-2206 largely abolished the inhibitory effects of HupA on PI-positive cell death, intracellular ROS accumulation, mitochondrial ROS production, and mitochondrial membrane depolarization.

Consistently, biochemical analyses demonstrated that MK-2206 significantly attenuated the HupA-mediated restoration of SOD activity and GSH content while reversing the reduction in MDA levels (Figure 7C). Immunoblot assays revealed that MK-2206 abrogated HupA-triggered Akt1 (Ser473) phosphorylation and MCL-1 upregulation, and eliminated the suppressive effect of HupA on supernatant HMGB1 release (Figure 7D,E). These findings indicate that pharmacological inhibition of AKT largely eliminates the antioxidant and mitochondrial protective effects of HupA, indicating that AKT signaling is an important contributor to HupA-mediated cardioprotection.

To further verify whether the inhibitory effect of HupA on HMGB1 release was closely associated with improved cell membrane integrity and reduced cellular injury, LDH release was measured as a canonical indicator of membrane damage (Figure S1). Consistent with the HMGB1 results, HupA pretreatment markedly reduced tBHP-induced LDH leakage and membrane disruption. However, MK-2206-mediated AKT inhibition completely reversed the membrane-protective effect of HupA, resulting in robust LDH release and severe cellular damage under oxidative stress. Collectively, these phenotypic, biochemical, and molecular observations indicate that pharmacological inhibition of AKT largely eliminates the antioxidant, mitochondrial-protective, and membrane-stabilizing effects of HupA. These findings indicate that AKT signaling contributes to HupA-mediated cardioprotection, and that the suppression of HMGB1 release by HupA is associated with AKT-dependent membrane protection and reduced cell injury.

## 4. Discussion

Oxidative stress is a major driving force in the development of myocardial injury [25]. Excessive ROS production disrupts redox homeostasis, impairs mitochondrial function, and activates multiple forms of cardiomyoblast death [23]. Therefore, identifying compounds capable of counteracting oxidative stress remains an important strategy for cardioprotection [26,27,28,29]. In the present study, we integrated network pharmacology, molecular docking, molecular dynamics simulation, and in vitro experimental validation to investigate the cardioprotective mechanisms of Huperzine A (HupA). Our findings identify AKT1 as a candidate molecular target involved in HupA-mediated cardioprotection and indicate that HupA attenuated tBHP-induced cardiomyoblast injury, accompanied by enhanced AKT1 activation, increased MCL-1 expression, and reduced HMGB1 release.

Network pharmacology analysis revealed that AKT1 occupied a central position in both the disease-associated and HupA-related target networks. AKT1 consistently ranked among the top hub genes identified by multiple network topology algorithms and was involved in several significantly enriched signaling pathways, including the PI3K/AKT pathway [30,31]. These findings suggest that AKT1 may represent an important molecular node linking HupA to the regulation of myocardial injury. Previous studies have demonstrated that activation of AKT1 signaling promotes cardiomyoblast survival and limits myocardial damage under oxidative stress and ischemic conditions. Therefore, the identification of AKT1 as a candidate target is consistent with the established cardioprotective role of this pathway.

To further investigate the potential association between HupA and AKT1, cellular thermal shift assay (CETSA), molecular docking and molecular dynamics analyses were performed. Consistent with the CETSA results showing that HupA increased the thermal stability of AKT1 protein in H9c2 cardiomyoblasts, molecular docking predicted a favorable interaction between HupA and AKT1, while MD simulations indicated that the predicted HupA–AKT1 complex remained relatively stable during a 100 ns simulation period. RMSD, RMSF, buried SASA, hydrogen-bond analysis, and free-energy landscape analysis collectively indicated that HupA maintained a stable interaction pattern with AKT1 during the simulation. MM/PBSA calculations further supported the energetic stability of the complex. Collectively, these findings provide complementary computational and cellular evidence supporting AKT1 as a potential molecular target of HupA and are consistent with cellular target engagement. While the CETSA results indicate that HupA engages AKT1 within cells and enhances its thermal stability, this assay primarily reflects target engagement rather than quantitative binding affinity or a defined binding site. The functional relevance of AKT1 engagement is supported by the observed restoration of AKT1 phosphorylation and downstream signaling under oxidative stress. Further studies employing biophysical approaches, such as surface plasmon resonance or isothermal titration calorimetry, and site-directed mutagenesis would help to define the binding affinity and precise binding interface of HupA with AKT1. Given that AKT1 signaling is a central regulator of cardiomyoblast survival under oxidative stress, we next investigated whether AKT1 activation contributes to the protective effects of HupA. Although computational analysis predicted a stable interaction between HupA and AKT1, the functional data demonstrated enhanced AKT1 phosphorylation rather than inhibition of AKT1 activity. Therefore, the potential association of HupA with AKT1 may contribute to the regulation of AKT1 signaling. Given the established role of AKT1 signaling in maintaining cardiomyoblast survival and cellular redox homeostasis, the enhanced AKT1 phosphorylation observed following HupA treatment may contribute to the attenuation of oxidative stress and cardiomyoblast injury observed in this study.

Oxidative stress-induced cardiomyoblast death is a major contributor to myocardial injury [32]. In the present study, tBHP exposure markedly reduced H9c2 cell viability and increased cell death, whereas HupA treatment significantly alleviated these effects. Importantly, HupA restored AKT1 phosphorylation suppressed by tBHP. AKT1 activation is known to regulate multiple downstream survival pathways, including members of the BCL-2 family. Consistent with this mechanism, HupA treatment increased the expression of the anti-apoptotic protein MCL-1. MCL-1 plays an essential role in maintaining mitochondrial integrity and preventing stress-induced cell death [33]. Beyond its anti-apoptotic function, MCL-1 is also essential for maintaining mitochondrial cristae architecture and respiratory function, thereby limiting mitochondrial ROS generation [29]. Therefore, AKT-associated signaling may contribute to the cytoprotective effects of HupA observed in this study.

Another notable finding was the inhibitory effect of HupA on HMGB1 release. HMGB1 is a representative damage-associated molecular pattern (DAMP) that is released from injured or stressed cells and subsequently amplifies inflammatory responses [34]. Elevated extracellular HMGB1 levels have been implicated in myocardial ischemia–reperfusion injury, heart failure, and adverse cardiac remodeling [35]. In the current study, tBHP-induced oxidative stress promoted HMGB1 release into the culture supernatant [36], whereas HupA significantly reduced extracellular HMGB1 levels. To further consolidate the underlying mechanism, LDH release was assessed as a reliable indicator of cell membrane damage and cellular injury. Collectively, our HMGB1 and LDH detection results support that HupA-mediated inhibition of HMGB1 extracellular release is tightly associated with its protective effect against oxidative stress-triggered cardiomyoblast membrane damage and cellular injury.

Given that AKT activation has been reported to preserve cellular integrity and limit DAMP release under stress conditions, the reduction in HMGB1 release observed after HupA treatment may be secondary to improved cell survival and enhanced cellular resistance to oxidative injury. Previous studies have demonstrated that HupA possesses potent antioxidant properties in neuronal and inflammatory disease models, where it reduces ROS accumulation and attenuates oxidative damage [37]. The present study extends these observations to cardiomyoblasts and suggests that AKT1 signaling may represent an important molecular mechanism underlying the antioxidant activity of HupA in the heart.

Notably, HupA also effectively reduced intracellular and mitochondrial ROS accumulation, restored mitochondrial membrane potential, and improved the cellular antioxidant capacity by decreasing MDA levels while increasing SOD and GSH activities. These findings indicate that HupA alleviates oxidative stress and preserves mitochondrial function in cardiomyoblasts. Consistent with previous studies demonstrating the antioxidant properties of HupA in neurological and inflammatory disorders [38], our findings further support a protective role of HupA against oxidative stress-induced cardiomyoblast injury potentially involving AKT-associated signaling.

To further verify the involvement of AKT1 signaling, the AKT allosteric inhibitor MK-2206 was used to pharmacologically block AKT1 activation [38,39]. MK-2206 markedly attenuated the protective effects of HupA, as evidenced by increased intracellular and mitochondrial ROS accumulation. These findings provide functional evidence supporting the involvement of AKT signaling for the antioxidant and protective effects of HupA in H9c2 rat cardiomyoblasts.

Several limitations of this study should be acknowledged. First, the experimental validation was performed primarily in the H9c2 rat cardiomyoblast cell line using a preventive treatment paradigm. Therefore, the present findings cannot be directly extrapolated to primary cardiomyoblasts, human cardiomyoblasts, or therapeutic settings. Further validation in primary and human cardiomyoblast models will be necessary to determine the broader relevance of the observed cardioprotective effects of HupA. Second, the observed changes in MCL-1 expression and extracellular HMGB1 release were not validated using specific genetic loss-of-function or gain-of-function approaches. Therefore, further studies using genetic pathway models, including targeted manipulation of AKT1, MCL-1, or HMGB1, are warranted to clarify the causal contribution of these molecules to the protective effects of HupA. Finally, the concentration of HupA used in the in vitro experiments may not precisely reflect the levels achievable in vivo, and the pharmacokinetic properties and pharmacological relevance of HupA warrant further investigation. Moreover, the cardioprotective efficacy and mechanistic relevance of HupA require confirmation in in vivo models of myocardial injury.

## 5. Conclusions

In summary, this study integrated computational prediction and experimental validation to demonstrate that HupA attenuates oxidative stress-induced H9c2 cardiomyoblast injury, potentially involving AKT-associated signaling. HupA reduced intracellular and mitochondrial ROS, preserved mitochondrial membrane potential, suppressed HMGB1 release, and enhanced cellular antioxidant capacity. These findings suggest that HupA merits further investigation as a potential cardioprotective agent.

## Figures and Tables

**Table 1:** The number of targets collected by each database.

Keyword	Database	Number of Targets	Actual Selection	Total	Union
Cardiac/Heart injury	CTD	32,432	Top2000		6180
	OMIM	137/137	274	3766	
	Genecards	9673/12,915	Top2000		
Heart failure	CTD	112,840	Top2000		
	OMIM	637	637	3592	
	Genecards	18,023	Top2000		
Myocardial injury/damage	CTD	24,234	Top2000		
	OMIM	34/78	112	3633	
	Genecards	5944/6676	Top2000		
MIRI	CTD	/	/		
	OMIM	44	44	2035	
	Genecards	2168	Top2000		

**Table 2:** Binding free energy analysis of AKT-HupA complexes (kcal/mol).

Energy Contributions	AKT-HupA
ΔEvdw (kcal/mol)	−125.34 ± 6.16
ΔEele (kcal/mol)	−28.20 ± 2.18
ΔEpol (kcal/mol)	102.37 ± 7.56
ΔEnonpol (kcal/mol)	−17.98 ± 0.27
ΔEMMPBSA (kcal/mol)	−69.16 ± 8.63
−TΔS (kcal/mol)	9.56 ± 1.43
ΔGbind * (kcal/mol)	−59.60 ± 9.86

* ΔGbind = ΔEvdw + ΔEele + ΔEpol + ΔEnonpol-TΔS.

## Data Availability

The original contributions presented in this study are included in the article/Supplementary Material. Further inquiries can be directed to the corresponding author(s).

## Supplementary Materials

The following supporting information can be downloaded at: https://www.mdpi.com/article/10.3390/antiox15101333/s1, Table S1: Common targets identified from the cardiac injury-related databases using the keywords “Cardiac/Heart injury” “Heart injury” “Myocardial injury/damage” and “MIRI”. Table S2: 21 common elements in “Disease” and “HupA”. Table S3: GO enrichment-BP. Table S4: GO enrichment-CC. Table S5: GO enrichment-MF. Table S6: KEGG Pathway. Table S7: Summary of statistical analyses for *p*-AKT1/AKT1, MCL-1, and HMGB1 in H9c2 cells. Figure S1: Effects of HupA on cell viability and tBHP-induced injury in H9c2. Figure S2: Molecular dynamics simulations of the HupA-AKT1 complex. Figure S3: AKT1 thermal melting curves from three independent biological replicates of the cellular thermal shift assay (CETSA).

## Abbreviations

The following abbreviations are used in this manuscript:

AKT	protein kinase B
GSH	glutathione
HMGB1	high mobility group box 1
HupA	Huperzine A
MDA	malondialdehyde
MCL-1	myeloid cell leukemia-1
ROS	reactive oxygen species

## References

[1] Cui J., Chen Y., Yang Q., Zhao P., Yang M., Wang X., Mang G., Yan X., Wang D., Tong Z. (2024). Protosappanin A protects DOX-induced myocardial injury and cardiac dysfunction by targeting ACSL4/FTH1 axis-dependent ferroptosis. Adv. Sci..

[2] Zhou H., Li C., Fatima S., Qiao L., Lu D., Li W. (2026). 3′-Methoxypuerarin ameliorates myocardial ischemia/reperfusion-induced pyroptosis via regulating IGF2BP1/m6A/NLRP3 pathway. Antioxid. Redox Signal..

[3] Wu Z., Huang N., Li C., Lin M., Chen Z., Li W., Zhou H. (2025). β-Sitosterol suppresses NLRP3 inflammasome activation and pyroptosis in myocardial ischemia/reperfusion injury via inhibition of PPARγ2. Int. Immunopharmacol..

[4] Juan C.A., Perez de la Lastra J.M., Plou F.J., Perez-Lebeña E. (2021). The Chemistry of Reactive Oxygen Species (ROS) Revisited: Outlining Their Role in Biological Macromolecules (DNA, Lipids and Proteins) and Induced Pathologies. Int. J. Mol. Sci..

[5] Zweck E., Piel S., Schmidt J.W., Scheiber D., Schon M., Kahl S., Burkart V., Dewidar B., Remus R., Chadt A. (2025). Impaired mitochondrial ketone body oxidation in insulin resistant states. EBioMedicine.

[6] Cao J., Chen X., Chen L., Lu Y., Wu Y., Deng A., Pan F., Huang H., Liu Y., Li Y. (2025). DHODH-mediated mitochondrial redox homeostasis: A novel ferroptosis regulator. Redox Biol..

[7] Weber D., Thimm J., Seiz T., Kochlik B., Raupbach J., Burkle A., Grune T., Gruber M., Moreno-Villanueva M. (2025). Association between redox biomarkers, DNA damage and aerobic capacity. Redox Biol..

[8] Bolduc J.A., Collins J.A., Loeser R.F. (2019). Reactive oxygen species, aging and cartilage homeostasis. Free Radic. Biol. Med..

[9] Hu J., Bao X., Ting M., Tao Y., Li R., Fu G., Qiu F., Zhao J., Zhang W. (2026). Precision targeting of FDX1-mediated cuproptosis. Theranostics.

[10] An J., Han C., Jiang Y., Shi J., Li H., Wang C., Huang J., Xu S., Ni J., Cao Y. (2026). Sam68 exacerbates pathological cardiac hypertrophy. Circulation.

[11] Li X., Chen X., Yang F.Y., Shu T., Jiang L., He B., Tang M., Fang D., Jose P.A., Han Y. (2025). Effect of TSPO on LPS-induced cardiac dysfunction. J. Adv. Res..

[12] Guan X., Li H., Zhang L., Zhi H. (2025). Mechanisms of mitochondrial damage-associated molecular patterns associated with inflammatory response in cardiovascular diseases. Inflamm. Res..

[13] Qin W., Cao L., Massey I.Y. (2021). Role of PI3K/Akt signaling in cardiac fibrosis. Mol. Cell. Biochem..

[14] Yang T.T., Zhou L.H., Gu L.F., Qian L.L., Bao Y.L., Jing P., Sun J.T., Du C., Shan T.K., Wang S.B. (2025). CHK1 attenuates cardiac dysfunction. Metabolism.

[15] Liu H., Liu W., Qiu H., Zou D., Cai H., Chen Q., Zheng C., Xu D. (2020). Salvianolic acid B protects against myocardial ischaemia-reperfusion injury in rats via inhibiting high mobility group box 1 protein expression through the PI3K/Akt signalling pathway. Naunyn Schmiedeberg’s Arch. Pharmacol..

[16] Takahashi T., Shishido T., Kinoshita D., Watanabe K., Toshima T., Sugai T., Narumi T., Otaki Y., Tamura H., Nishiyama S. (2019). Cardiac Nuclear High-Mobility Group Box 1 Ameliorates Pathological Cardiac Hypertrophy by Inhibiting DNA Damage Response. JACC Basic Transl. Sci..

[17] Chen X., Zhang Y., Cao Z., Wang Y., Liao M., Guan Y., Zhu C., Wang W., Huang W., Li W. (2024). Huperzine A targets Apolipoprotein E: A potential therapeutic drug for diabetic nephropathy based on omics analysis. Pharmacol. Res..

[18] Wang R., Tang X.C. (2005). Neuroprotective effects of huperzine A. A natural cholinesterase inhibitor for the treatment of Alzheimer’s disease. Neuro-Signals.

[19] Rispoli V., Ragusa S., Nistico R., Marra R., Russo E., Leo A., Felicitá V., Rotiroti D. (2013). Huperzine a restores cortico-hippocampal functional connectivity after bilateral AMPA lesion of the nucleus basalis of meynert. J. Alzheimers Dis..

[20] Le T.T.M., Trinh H.T.T., Pham H.T., Nguyen D.T., Do G.H., Phan H.T., Le T.D., Quach N.T., Phi T.Q., Chu H.H. (2025). Lycopodiaceae herb from Vietnam as a promising medicinal source of natural hupezine and novel huperzine-producing endophytic fungi. Biotechnol. Lett..

[21] Su J., Yang M. (2015). Huperzine A production by *Paecilomyces tenuis* YS-13, an endophytic fungus isolated from *Huperzia serrata*. Nat. Prod. Res..

[22] Zhang C., Li M., Xie W., You C., Wang T., Fu F. (2023). Administration of Huperzine A microspheres ameliorates myocardial ischemic injury via α7nAChR-dependent JAK2/STAT3 signaling pathway. Eur. J. Pharmacol..

[23] Chen Y., Guo X., Zeng Y., Mo X., Hong S., He H., Li J., Fatima S., Liu Q. (2023). Oxidative stress induces mitochondrial iron overload and ferroptotic cell death. Sci. Rep..

[24] Zheng K., Zhang Q., Lin G., Li Y., Sheng Z., Wang J., Chen L., Lu H.H. (2017). Activation of Akt by SC79 protects myocardiocytes from oxygen and glucose deprivation (OGD)/re-oxygenation. Oncotarget.

[25] Mei S., Hong L., Cai X., Xiao B., Zhang P., Shao L. (2019). Oxidative stress injury in doxorubicin-induced cardiotoxicity. Toxicol. Lett..

[26] Ali S.S., Ahmad W.A.N.W., Budin S.B., Zainalabidin S. (2020). Implication of dietary phenolic acids on inflammation in cardiovascular disease. Rev. Cardiovasc. Med..

[27] Chen G., Wei M., Zhao C.Y., Zhang A.Y., Su J.B., Ni Z.R., Cai W.W., Hou B., Du B., Liu M.H. (2025). Phillygenin ameliorates myocardial ischemia-reperfusion injury by inhibiting cuproptosis via the autophagy-lysosome degradation of CTR1. Free Radic. Biol. Med..

[28] Sishu N.K., Selvaraj C.I. (2025). Cichoriin, a coumarin glycoside with antidiabetic effect and cardioprotective activity against H_2_O_2_ induced oxidative stress in H9c2 cells: In-vitro and in-silico study. Free Radic. Biol. Med..

[29] Bermudez-Oria A., Godoy E., Perez V., Musci-Ferrari C., Donato M., Fernandez-Bolaños J., Zaobornyj T., D’Annunzio V. (2025). Unveiling the Cardioprotective Potential of Hydroxytyrosol: Insights from an Acute Myocardial Infarction Model. Antioxidants.

[30] Ersahin T., Tuncbag N., Cetin-Atalay R. (2015). The PI3K/AKT/mTOR interactive pathway. Mol. BioSyst..

[31] DeBosch B., Treskov I., Lupu T.S., Weinheimer C., Kovacs A., Courtois M., Muslin A.J. (2006). Akt1 is required for physiological cardiac growth. Circulation.

[32] Guo X., Hong S., He H., Zeng Y., Chen Y., Mo X., Li J., Li L., Steinmetz R., Liu Q. (2020). NFκB promotes oxidative stress-induced necrosis and ischemia/reperfusion injury by inhibiting Nrf2-ARE pathway. Free Radic. Biol. Med..

[33] Morciano G., Giorgi C., Balestra D., Marchi S., Perrone D., Pinotti M., Pinton P. (2016). Mcl-1 involvement in mitochondrial dynamics is associated with apoptotic cell death. Mol. Biol. Cell.

[34] Ding X., Li S., Zhu L. (2021). Potential effects of HMGB1 on viral replication and virus infection-induced inflammatory responses: A promising therapeutic target for virus infection-induced inflammatory diseases. Cytokine Growth Factor Rev..

[35] Lu H., Zhang Z., Barnie P.A., Su Z. (2019). Dual faced HMGB1 plays multiple roles in cardiomyocyte senescence and cardiac inflammatory injury. Cytokine Growth Factor Rev..

[36] Hu T., Cui P., Zhao X., Wang W., Ma Y., Hu X., Zhang H., Fan Z., Wang D., Huang Q. (2026). UCHL3 drives ferroptosis in nucleus pulposus cells by deubiquitinating HMGB1 to activate NCOA4-mediated ferritinophagy. Autophagy.

[37] Mohseni-Moghaddam P., Sadr S.S., Roghani M., Arabzadeh S., Khamse S., Zamani E., Hosseini M., Moradi F. (2019). Huperzine A ameliorates cognitive dysfunction and neuroinflammation in kainic acid-induced epileptic rats by antioxidant activity and NLRP3/caspase-1 pathway inhibition. Clin. Exp. Pharmacol. Physiol..

[38] Zhang H., Wang D., Sun J., Wang Y., Wu S., Wang J. (2022). Huperzine-A Improved Animal Behavior in Cuprizone-Induced Mouse Model by Alleviating Demyelination and Neuroinflammation. Int. J. Mol. Sci..

[39] Xu F., Zhao X., Zhang J., Shen C. (2024). Halofuginone ameliorates the susceptibility to atrial fibrillation by activating the PI3K/Akt signaling pathway. J. Mol. Histol..

