# Supplementary material for: Huperzine A Attenuates Oxidative Stress-Induced H9c2 Cardiomyoblast Injury Through AKT-Associated Survival Signaling
Source: Antioxidants (Basel). 2026 Oct 15;15(10):1333. doi: 10.3390/antiox15101333 (PMC13701573; doi:10.3390/antiox15101333)
Supplement: Supplementary file 1 [file antioxidants-15-01333-s001.zip › antioxidants-4431985-supplementary.pdf]

Supplementary Data

Table S1 Common targets identified from the cardiac injury-related databases using the keywords "Cardiac/Heart injury" "Heart injury" "Myocardial injury/damage" and "MIRI"

NO.	Gene Symbol
1	TP53
2	ACE
3	MIAT
4	PARP1
5	H2AX
6	IL6
7	AKT1
8	TNNT2
9	TNNI3
10	GADD45A
11	H19
12	TNF
13	MYH7
14	CRP
15	NPPA
16	SCN5A
17	CASP3
18	ESR1
19	APOB
20	PCSK9
21	TGFB1
22	OLR1
23	SOD1
24	NOS3
25	MYBPC3
26	SERPINE1
27	XRCC1
28	DDIT3
29	LDLR
30	NPPB
31	MB
32	MDM2
33	PLAT
34	APOA1
35	IL10
36	SIRT1
37	APOE
38	MIR21
39	VEGFA

40	MYZAP
41	TLR4
42	PCNA
43	THBD
44	GADD45G
45	KCNQ1
46	DDIT4
47	APEX1
48	CDKN1A
49	HMOX1
50	CAT
51	ABCA1
52	TTR
53	IL1B
54	LPL
55	MMP9
56	DSP
57	NLRP3
58	CCL2
59	GATA4
60	PKP2
61	HIF1A
62	ADIPOQ
63	PTGS2
64	BAX
65	STAT3
66	MTOR
67	GCLC
68	BCL2
69	CDKN2A
70	SOD2
71	MYH6
72	TPM1
73	NFE2L2
74	F7
75	MPO
76	F2
77	PTEN
78	VWF
79	CXCL8
80	ALB
81	BAG3
82	CBS
83	PTPN11

84	HMGB1
85	MMP2
86	DMD
87	MMP3
88	CAV3
89	IFNG
90	DES
91	MYL3
92	GCLM
93	CXCL12
94	ALDH2
95	CYCS
96	HRAS
97	EDN1
98	CTNNB1
99	JAK2
100	ICAM1
101	FASLG
102	CACNA1C
103	SMAD3
104	MYC
105	JUP
106	CPT2
107	VCL
108	GJA1
109	F3
110	AGT
111	LGALS3
112	ELN
113	RAF1
114	KRAS
115	FBN1
116	CST3
117	GDF15
118	PPARG
119	CRYAB
120	CKB
121	CASP9
122	CCND1
123	CSRP3
124	SELE
125	XDH
126	ACTN2
127	CFH

128	MMP1
129	MAPK14
130	ACE2
131	IGF1
132	FAS
133	AGER
134	NOS2
135	NAGLU
136	LCN2
137	CDK1
138	CALM1
139	CKM
140	IL1RN
141	EGFR
142	NOTCH1
143	TGFB3
144	FXN
145	NFKB1
146	CASP8
147	MAP2K1
148	HSPD1
149	GSK3B
150	ACADVL
151	IL18
152	PML
153	EP300
154	ACTA2
155	CXCR4
156	AARS1
157	SDHA
158	IL1A
159	ENG
160	GSTP1
161	TRDN
162	TNFRSF1A
163	SLC25A4
164	FGA
165	PPARGC1A
166	MAPK1
167	HP
168	PLN
169	CD36
170	FEN1
171	COL3A1

172	TIMP1
173	CCN2
174	ANKRD1
175	HMGCR
176	MGMT
177	CLU
178	HGF
179	CALM3
180	SMAD4
181	CP
182	CYBA
183	SQSTM1
184	CREBBP
185	NDUFS4
186	HSPB1
187	DNM1L
188	HSPA5
189	SP1
190	KCND3
191	GPT
192	TF
193	GLB1
194	CDH2
195	GSTM1
196	CYBB
197	GBE1
198	FANCA
199	BCL2L1
200	HSPA8
201	ACADM
202	NOTCH3
203	TOP2A
204	ADRB2
205	PRKCD
206	APLN
207	PPP1R15A
208	COL1A1
209	MIR34A
210	PECAM1
211	PNPLA2
212	COL4A1
213	FOXO3
214	HIPK2
215	FHL1

216	FGF2
217	CD40LG
218	MFN2
219	IL17A
220	MAPT
221	STAT1
222	MAPK8
223	SMAD2
224	CTSD
225	KLF4
226	VCAM1
227	IL4
228	CACNB2
229	PTGS1
230	RB1
231	CPT1A
232	MIR155
233	GSR
234	SERPINA1
235	CD14
236	GSTT1
237	SLC25A20
238	CYP1B1
239	MAP2K2
240	ITGAM
241	CFTR
242	G6PD
243	DLD
244	GFAP
245	CCNB1
246	IL33
247	SLC2A1
248	EPHX1
249	ITGA2
250	FOXO1
251	TSFM
252	YWHAE
253	JUN
254	HADH
255	HDAC1
256	FLT1
257	SPP1
258	ADM
259	TGFBR2

260	PPARA
261	AVP
262	DNMT1
263	FN1
264	AIFM1
265	MME
266	PLG
267	KDR
268	LEP
269	NDUFAF2
270	CASP1
271	GPX1
272	PINK1
273	APP
274	NPM1
275	NGF
276	NOX4
277	NDUFV1
278	NFKBIA
279	SOD3
280	NDUFS1
281	TNFRSF11B
282	ITGB1
283	PLA2G7
284	TFAM
285	MIF
286	TGFBR1
287	CCL5
288	NDUFS3
289	CSF3
290	HSPA4
291	TH
292	MYH9
293	SRC
294	CREB1
295	CCR2
296	INSR
297	MIB1
298	PTPRC
299	PTX3
300	AHCY
301	ALPK3
302	IL6R
303	TGFB2

304	LOX
305	EGR1
306	HSD17B10
307	ETFDH
308	UBC
309	NCF1
310	FBXO32
311	XIAP
312	DNAJC19
313	ACADS
314	IRF1
315	BID
316	NDUFS2
317	C3
318	ATP2A2
319	DPP4
320	IRS1
321	CYP1A1
322	JAG1
323	NDUFA1
324	MIR146A
325	ATG5
326	EZH2
327	MAPK3
328	CX3CR1
329	NDUFV2
330	CHKA
331	RIPK1
332	NDUFS8
333	COMT
334	TSC2
335	ACTB
336	BECN1
337	CXCL10
338	PRKCA
339	CALCA
340	S100A9
341	PDE5A
342	COL4A2
343	ATP5F1A
344	PDGFRB
345	CCND2
346	HSP90AA1
347	IL2RA

348	KIT
349	LCAT
350	MYD88
351	CYP2E1
352	IL2
353	HDAC9
354	GHRL
355	KEAP1
356	MMP13
357	ADA
358	ATP5F1E
359	TXN
360	NDUFS6
361	SIRT3
362	IGFBP1
363	TIMP2
364	MCCC2
365	SDHB
366	NQO1
367	PRKAA2
368	UQCRFS1
369	GPX3
370	HAMP
371	RELA
372	APAF1
373	PDGFRA
374	ECHS1
375	PGK1
376	GGT1
377	S100A8
378	ATP1A1
379	FOS
380	BSG
381	PLEC
382	MEF2C
383	HNRNPA1
384	FABP7
385	CDH5
386	PSMB8
387	CALR
388	MIR132
389	MRPS22
390	CDKN1C
391	NDUFAF1

392	SPRED2
393	CCL3
394	BDNF
395	PLAUR
396	BMP4
397	DNMT3A
398	VIM
399	SLC2A4
400	EDNRA
401	ATF2
402	ETFA
403	NDUFB8
404	UCP2
405	IL6ST
406	COX7B
407	MYLK
408	NDUFA11
409	MCL1
410	DCN
411	THBS2
412	HSPA1A
413	NDUFA10
414	CYP1A2
415	TIMP3
416	MIR122
417	MIR27A
418	LAMP1
419	PRKAA1
420	IKBKB
421	RASSF1
422	IGF1R
423	ACAD8
424	ATP7B
425	NDUFA2
426	PSMC5
427	VTN
428	ITGB2
429	ANXA2
430	HAVCR1
431	ERBB4
432	GUSB
433	DKK1
434	MAP1LC3B
435	CHUK

436	NPPC
437	MAT2A
438	YWHAG
439	IGF2
440	CDC42
441	ACHE
442	EIF2AK3
443	GRN
444	SESN2
445	SOCS3
446	GHR
447	PIK3R1
448	PLAU
449	BNIP3
450	CASP2
451	IGFBP3
452	ITPA
453	GNAI2
454	TLR3
455	THPO
456	TJP1
457	KRT18
458	TRAF6
459	KLHL24
460	TUBB3
461	ARG1
462	MGP
463	CASP7
464	EDNRB
465	PSMC3
466	SLC2A10
467	SUCLA2
468	HAND2
469	SHH
470	HSPG2
471	NDRG1
472	MIR423
473	CYP7B1
474	ALDOA
475	ATF3
476	MAP1LC3A
477	PTK2
478	ABCG2
479	COL6A2

480	KL
481	LDHA
482	COL5A2
483	AGRN
484	PYGM
485	TFRC
486	PRL
487	AR
488	ACO2
489	CYP27B1
490	MLKL
491	ULK1
492	TXNIP
493	SLC8A1
494	CS
495	NR1H3
496	NAMPT
497	COX4I1
498	VCAN
499	GPX4
500	G6PC1
501	CSF2
502	PKM
503	CAMK2D
504	MAPK10
505	SOX9
506	RAC1
507	LIF
508	ATF4
509	TUFM
510	FABP4
511	ACACA
512	IL1R1
513	HJV
514	EGF
515	CBX1
516	BTG2
517	PRDX1
518	VDR
519	YWHAQ
520	MBP
521	PDHA1
522	AKT3
523	MIR19A

524	PIEZO1
525	GREM1
526	MAPK9
527	AHR
528	COL6A1
529	LYN
530	DIABLO
531	CXCL1
532	GLUL
533	TNC
534	IL12A
535	HMGA1
536	ENO1
537	GPT2
538	TGM2
539	SLC5A1
540	CTSB
541	SLC2A2
542	PIGA
543	SREBF1
544	FADD
545	EIF2S1
546	CYP7A1
547	ERN1
548	MICU1
549	KIF1B
550	FTH1
551	CTNNA1
552	TKT
553	TRIM21
554	TNXB
555	PPP2CA
556	MAF
557	CTSL
558	PRKCE
559	PAX6
560	NES
561	ESM1
562	DNMT3B
563	ATF6
564	SLC40A1
565	LAMB1
566	ACOX1
567	TPI1

568	LITAF
569	VEGFC
570	COL2A1
571	CTSK
572	MAP3K1
573	RUNX2
574	LBP
575	COQ9
576	PLA2G2A
577	PFKM
578	CFB
579	BGLAP
580	PRDX2
581	GSDMD
582	MAP3K5
583	CYP19A1
584	AQP1
585	AKT2
586	MIR342
587	ETS1
588	CASP12
589	EPHX2
590	FASN
591	INS1
592	ABCC2
593	LEPR
594	CDH1
595	SLC22A1
596	ABCB11
597	CXCL2
598	COX1
599	RPS6KB1
600	LHB
601	FOSB
602	STAR
603	PDK4
604	CD68
605	DUSP1
606	JUNB
607	PPP1R12A
608	FOSL1
609	GOT1
610	OCLN
611	TXNRD1

612	PDK1
613	ALPL
614	RPS6
615	ABCC3
616	PCK1
617	P4HA1
618	ANGPTL4
619	MAOA
620	MT2A
621	ITGAV
622	SCD1
623	HSP90B1
624	HOMER1
625	XBP1
626	CEBPB
627	SGK1
628	NEDD9
629	BMAL1
630	MT3
631	NCF2
632	MT1A
633	PRKCB
634	ACAA2
635	S100A6
636	MAOB
637	FSHB
638	NTRK2
639	LIPE
640	CIDEA
641	ABCG1
642	IGFBP2
643	ID2
644	IRS2
645	NFIL3
646	HSPA1B
647	SERPINH1
648	ABCC1
649	DUSP5
650	CCNE1
651	ATP5F1B
652	ITGA3
653	GAP43
654	PRDX3
655	SLC7A11

656	ACACB
657	PMAIP1
658	TRP53
659	BIRC3
660	NUPR1
661	STAT2
662	CEBPD
663	ECE1
664	HSPH1
665	NR4A1
666	ALDH1A1
667	NR1H4
668	PDIA3
669	ANXA1
670	TJP2
671	AQP3
672	HBEGF
673	ODC1
674	MAFF
675	ERCC1
676	NR0B2
677	NR1D1
678	PLIN2
679	TCF4
680	HMGCS2
681	ABCB4
682	SLC2A3
683	PYGL
684	NFKBIZ
685	HSPA2
686	SREBF2
687	TGFA
688	CFLAR
689	POR
690	TGIF1
691	CYP2B10
692	ADRA1B
693	ROCK2
694	SLPI
695	GCNT2
696	CREM
697	PPARGC1B
698	CD74
699	GPAM

700	ID1
701	HK2
702	CRY1
703	CYP4A14
704	IFI44
705	ASNS
706	LDHB
707	ABCC4
708	GSTA4
709	PMP22
710	IREB2
711	NEDD4L
712	AIF1
713	MT1
714	TK1
715	WSB1
716	CASP6
717	NFKBIB
718	SPHK1
719	UCP3
720	SNAI1
721	GPD1
722	CCN1
723	GSTM2
724	GSTA1
725	SLCO1A1
726	PARP12
727	CDK5R1
728	HSD3B1
729	DBN1
730	AFP
731	GOT2
732	HSPA1L
733	PRKAR2B
734	CYP11A1
735	MSMO1
736	ATP1B1
737	PRDX6
738	CFL1
739	FOXP1
740	STC2
741	TRIB3
742	FOXA2
743	OGDH

744	VLDLR
745	EIF5A
746	CXCL9
747	INHBB
748	RND1
749	ATOX1
750	USP2
751	CCNG2
752	BDKRB1
753	FIS1
754	ITPR3
755	DNAJB9
756	NR1I2
757	DNM1
758	A2M
759	PEBP1
760	PCK2
761	MGLL
762	IGFBP5
763	ZBTB16
764	AXIN2
765	SLC30A1
766	TNFRSF11A
767	RBP4
768	CD9
769	ID3
770	CAPN2
771	BCL3
772	CYP26B1
773	TUBA4A
774	CYP3A11
775	EEF1A1
776	PER1
777	ABHD2
778	XAF1
779	SERPINB2
780	DBP
781	BBS2
782	PCSK1
783	SMO
784	GSTA2
785	DNAJB4
786	LBH
787	SULT1A1

788	MGST1
789	DNAJA1
790	COXFA4
791	GDA
792	SERPINE2
793	CTH
794	SELENOP
795	IER3
796	TGFBI
797	ATP8B1
798	TNFAIP6
799	CITED2
800	SHMT1
801	COX6C
802	CPS1
803	COX7C
804	ALAS1
805	ASS1
806	HERPUD1
807	ASAP1
808	ACSL5
809	ARHGEF3
810	ERO1A
811	TFAP2A
812	PRKD1
813	AK4
814	INSIG1
815	DUSP4
816	CAMK2B
817	STAT6
818	ATP5PD
819	SRXN1
820	CANX
821	RGS2
822	CAMKK2
823	AURKA
824	RASD1
825	STC1
826	NFATC1
827	ENDOG
828	MKNK2
829	FOSL2
830	SQLE
831	IRF9

832	KRT8
833	DLAT
834	PSIP1
835	HNRNPH2
836	ZEB2
837	ATP6AP2
838	IGFBP7
839	ACSL4
840	KDM6A
841	PDE4A
842	MFGE8
843	ECM1
844	VASP
845	JARID2
846	MIR193B
847	NAGA
848	MEIS2
849	KLF2
850	UQCRC2
851	FARS2
852	DLK1
853	SEMA3E
854	FKBP5

Table S2 21 common elements in "Disease" and "HupA"

NO.	Gene Symbol
1	PARP1
2	AKT1
3	JAK2
4	GSK3B
5	PRKCD
6	EPHX1
7	KDR
8	SRC
9	DPP4
10	PDGFRB
11	PDGFRA
12	PRKAA1
13	CDC42
14	ACHE
15	CAMK2D
16	PRKCE
17	AKT2
18	EPHX2

19	MAOA
20	ROCK2
21	PRKD1

Table S3 GO enrichment-BP

Category	Term	Term	Count	%	PValue	Genes
BP	GO:0046777	protein autophosphorylation	7	33.3333333333333333	1.128509966866576E-9	PDGFRB, PDGFRA, GSK3B, KDR, AKT1, PRKD1, JAK2
	GO:0051897	positive regulation of phosphatidylinositol 3-kinase/protein kinase B signal transduction	8	38.095238095238095	1.385467551359862E-9	CDC42, PDGFRB, PDGFRA, SRC, KDR, AKT1, PRKD1, JAK2
	GO:0030335	positive regulation of cell migration	8	38.095238095238095	1.2421454785527976E-8	CDC42, PDGFRB, PDGFRA, ROCK2, AKT2, KDR, AKT1, JAK2
	GO:0032956	regulation of actin cytoskeleton organization	6	28.5714285714285714	7.14736294027266E-8	CDC42, PDGFRB, PDGFRA, ROCK2, PRKCE, PRKCD
	GO:0043410	positive regulation of MAPK cascade	7	33.3333333333333333	8.701902247792314E-8	CDC42, PDGFRB, PDGFRA, ROCK2, PRKCE, KDR, JAK2
	GO:0007165	signal transduction	11	52.3809523809525239	3.6617252849688886E-7	CDC42, PDGFRB, PRKAA1, ROCK2, SRC, AKT2, PRKCE, PRKCD, AKT1, PRKD1, JAK2
	GO:0035556	intracellular signal transduction	8	38.095238095238095	5.330764826372064E-7	GSK3B, SRC, AKT2, PRKCE, PRKCD, AKT1, PRKD1, JAK2
	GO:0043066	negative regulation of apoptotic process	8	38.095238095238095	7.913638206834253E-7	GSK3B, PRKAA1, SRC, AKT2, PRKCD, KDR, AKT1, JAK2
	GO:0018108	peptidyl-tyrosine phosphorylation	4	19.047619047619047	6.780859048433748E-6	PDGFRB, PDGFRA, SRC, KDR
	GO:0042307	positive regulation of protein import into nucleus	4	19.047619047619047	1.0859124240526498E-5	PRKCD, AKT1, PRKD1, JAK2

Table S4 GO enrichment-CC

Category	Term	Term	Count	%	PValue	Genes

	GO:0005829	cytosol	17	80.95238095238095	3.05912468923458 97E-6	PDGFRA, GSK3B, CAMK2D, PRKAA1, PARP1, MAOA, ROCK2, SRC, EPHX2, PRKCE, PRKCD, CDC42, AKT2, KDR, AKT1, PRKD1, JAK2
	GO:0005737	cytoplasm	17	80.95238095238095	3.239131796461908E-5	PDGFRB, PDGFRA, GSK3B, CAMK2D, PRKAA1, PARP1, ROCK2, SRC, EPHX2, PRKCE, PRKCD, CDC42, AKT2, KDR, AKT1, PRKD1, JAK2
	GO:0005911	cell-cell junction	5	23.80952380952380 7	4.913962245571755 6E-5	CDC42, SRC, PRKCD, AKT1, PRKD1
	GO:0005925	focal adhesion	6	28.57142857142857	5.662185500368067E-5	CDC42, PDGFRB, DPP4, GSK3B, SRC, JAK2
	GO:0005634	nucleus	16	76.19047619047619	1.682618706984641 8E-4	PDGFRB, PDGFRA, ACHE, GSK3B, CAMK2D, PRKAA1, PARP1, ROCK2, SRC, PRKCE, PRKCD, AKT2, KDR, AKT1, PRKD1, JAK2
CC	GO:0005794	Golgi apparatus	8	38.09523809523809 5	1.914971868200382E-4	PDGFRB, PDGFRA, GSK3B, ACHE, PRKCE, PRKCD, KDR, PRKD1
	GO:0005886	plasma membrane	15	71.42857142857143	1.993262333192226 3E-4	PDGFRB, PDGFRA, ACHE, GSK3B, ROCK2, SRC, PRKCE, PRKCD, CDC42, DPP4, AKT2, KDR, AKT1, PRKD1, JAK2
	GO:0016020	membrane	15	71.42857142857143	2.255974177342864 4E-4	PDGFRB, PDGFRA, ACHE, CAMK2D, PARP1, MAOA, SRC, PRKCE, PRKCD, CDC42, DPP4, KDR, AKT1, PRKD1, JAK2
	GO:0048471	perinuclear region of cytoplasm	6	28.57142857142857	6.512562868587893E-4	GSK3B, ACHE, SRC, PRKCE, PRKCD, PRKD1
	GO:0045121	membrane raft	4	19.04761904761904 7	0.001366949699093 4514	DPP4, SRC, KDR, JAK2
	GO:0098794	postsynapse	4	19.04761904761904 7	0.001949706007634 412	CDC42, GSK3B, AKT1, JAK2

Table S5 GO enrichment-MF

Category	Term	Term	Count	%	PValue	Genes
MF	GO:0004672	protein kinase activity	14	66.6666666666666666	2.50829338225653 37E-16	PDGFRB, PDGFRA, GSK3B, CAMK2D, PRKAA1, ROCK2, SRC, PRKCE, PRKCD, AKT2, KDR, AKT1, PRKD1, JAK2
	GO:0005524	ATP binding	14	66.6666666666666666	2.39076917920420 17E-10	PDGFRB, PDGFRA, GSK3B, CAMK2D, PRKAA1, ROCK2, SRC, PRKCE, PRKCD, AKT2, KDR, AKT1, PRKD1, JAK2
	GO:0106310	protein serine kinase activity	9	42.857142857142854	1.62265546227255 12E-9	GSK3B, CAMK2D, PRKAA1, ROCK2, AKT2, PRKCE, PRKCD, AKT1, PRKD1
	GO:0004674	protein serine/threonine kinase activity	9	42.857142857142854	4.26059699374178 5E-9	GSK3B, CAMK2D, PRKAA1, ROCK2, AKT2, PRKCE, PRKCD, AKT1, PRKD1
	GO:0004713	protein tyrosine kinase activity	6	28.57142857142857	2.45959639732346 5E-7	PDGFRB, PDGFRA, SRC, PRKCD, KDR, JAK2
	GO:0016301	kinase activity	6	28.57142857142857	6.70853991893402 2E-6	PDGFRB, GSK3B, SRC, AKT1, PRKD1, JAK2
	GO:0019901	protein kinase binding	7	33.33333333333333	7.48249404090132 2E-6	CDC42, PDGFRB, GSK3B, PARP1, PRKCD, AKT1, JAK2
	GO:0038085	vascular endothelial growth factor binding	3	14.285714285714285	1.01631234826545 15E-5	PDGFRB, PDGFRA, KDR
	GO:0004699	diacylglycerol-dependent, calcium-independent serine/threonine kinase activity	3	14.285714285714285	2.84037537096425 62E-5	PRKCE, PRKCD, PRKD1
	GO:0030546	signaling receptor activator activity	3	14.285714285714285	3.64964310100850 9E-5	SRC, PRKCE, JAK2

Table S6 KEGG Pathway

Category	Term	Count	%	PValue	Genes

KEGG_PATHWAY	hsa01521:EGFR tyrosine kinase inhibitor resistance	8	38.095238095238095	1.7880220157875487E-10	PDGFRB, PDGFRA, GSK3B, SRC, AKT2, KDR, AKT1, JAK2
	hsa04510:Focal adhesion	9	42.857142857142854	4.244672628143648E-9	CDC42, PDGFRB, PDGFRA, GSK3B, ROCK2, SRC, AKT2, KDR, AKT1
	hsa04518:Integrin signaling	8	38.095238095238095	1.824587482644501E-8	CDC42, PDGFRB, PDGFRA, GSK3B, SRC, AKT2, KDR, AKT1
	hsa04062:Chemokine signaling pathway	8	38.095238095238095	8.686830737826882E-8	CDC42, GSK3B, ROCK2, SRC, AKT2, PRKCD, AKT1, JAK2
	hsa04015:Rap1 signaling pathway	8	38.095238095238095	1.6537325446233324E-7	CDC42, PDGFRB, PDGFRA, SRC, AKT2, KDR, AKT1, PRKD1
	hsa05417:Lipid and atherosclerosis	8	38.095238095238095	1.8791433012053353E-7	CDC42, GSK3B, CAMK2D, ROCK2, SRC, AKT2, AKT1, JAK2
	hsa05131:Shigellosis	8	38.095238095238095	5.508824860553382E-7	CDC42, GSK3B, ROCK2, SRC, AKT2, PRKCE, PRKCD, AKT1
	hsa04933:AGE-RAGE signaling pathway in diabetic complications	6	28.57142857142857	1.79146601807136E-6	CDC42, AKT2, PRKCE, PRKCD, AKT1, JAK2
	hsa04931:Insulin resistance	6	28.57142857142857	2.6140928608091765E-6	GSK3B, PRKAA1, AKT2, PRKCE, PRKCD, AKT1
	hsa05205:Proteoglycans in cancer	7	33.33333333333333	2.963989667834574E-6	CDC42, CAMK2D, ROCK2, SRC, AKT2, KDR, AKT1
	hsa04722:Neurotrophin signaling pathway	6	28.57142857142857	4.201766886123207E-6	CDC42, GSK3B, CAMK2D, AKT2, PRKCD, AKT1
	hsa05208:Chemical carcinogenesis - reactive oxygen species	7	33.33333333333333	5.502535662162127E-6	SRC, AKT2, EPHX2, EPHX1, PRKCD, AKT1, PRKD1
	hsa04151:PI3K-Akt signaling pathway	8	38.095238095238095	6.058435848073405E-6	PDGFRB, PDGFRA, GSK3B, PRKAA1, AKT2, KDR, AKT1, JAK2
	hsa04810:Regulat	7	33.333	6.23935	CDC42, PDGFRB,

	ion of actin cytoskeleton		333333 33333	8153534 855E-6	PDGFRA, ROCK2, SRC, AKT2, AKT1
	hsa04370:VEGF signaling pathway	5	23.809 523809 523807	6.80333 0315315 4585E-6	CDC42, SRC, AKT2, KDR, AKT1
	hsa05200:Pathways in cancer	9	42.857 142857 142854	7.09952 7969437 738E-6	CDC42, PDGFRB, PDGFRA, GSK3B, CAMK2D, ROCK2, AKT2, AKT1, JAK2
	hsa05135:Yersinia infection	6	28.571 428571 42857	8.34164 3926235 741E-6	CDC42, GSK3B, ROCK2, SRC, AKT2, AKT1
	hsa04917:Prolactin signaling pathway	5	23.809 523809 523807	1.33532 7128622 143E-5	GSK3B, SRC, AKT2, AKT1, JAK2
	hsa05214:Glioma	5	23.809 523809 523807	1.75116 9912892 6157E-5	PDGFRB, PDGFRA, CAMK2D, AKT2, AKT1
	hsa04012:ErbB signaling pathway	5	23.809 523809 523807	2.85906 0966038 7043E-5	GSK3B, CAMK2D, SRC, AKT2, AKT1

Table S7 Summary of statistical analyses for p-AKT1/AKT1, MCL-1, and HMGB1 in H9c2 cells.

Analysis step	p-AKT1/AKT1	MCL-1	HMGB1
One-way ANOVA (all 7	F(6, 14) = 330.59, p <	F(6, 14) = 836.44, p	F(6, 14) = 3295.2, p

groups)	0.0001, η² = 0.993	< 0.0001, η² = 0.997	< 0.0001, η² = 0.999
Two-way factorial ANOVA: tBHP × HupA — HupA main effect	F(1, 8) = 110.84, p < 0.0001, partial η² = 0.933	F(1, 8) = 714.69, p < 0.0001, partial η² = 0.989	F(1, 8) = 16182.8, p < 0.0001, partial η² ≈ 1.000
— tBHP main effect	F(1, 8) = 362.35, p < 0.0001, partial η² = 0.978	F(1, 8) = 324.93, p < 0.0001, partial η² = 0.976	F(1, 8) = 16399.0, p < 0.0001, partial η² ≈ 1.000
— Interaction (tBHP × HupA)	F(1, 8) = 224.50, p < 0.0001, partial η² = 0.966	F(1, 8) = 909.71, p < 0.0001, partial η² = 0.991	F(1, 8) = 16227.5, p < 0.0001, partial η² ≈ 1.000
Two-way factorial ANOVA: tBHP × MK-2206 — MK-2206 main effect	F(1, 8) = 31.23, p = 0.0005, partial η² = 0.796	F(1, 8) = 0.50, p = 0.501, partial η² = 0.059 (ns)	F(1, 8) = 23400.2, p < 0.0001, partial η² ≈ 1.000
— tBHP main effect	F(1, 8) = 1160.46, p < 0.0001, partial η² = 0.993	F(1, 8) = 1955.44, p < 0.0001, partial η² = 0.996	F(1, 8) = 2.52, p = 0.151, partial η² = 0.240
— Interaction (tBHP × MK-2206)	F(1, 8) = 39.35, p = 0.0002, partial η² = 0.831	F(1, 8) = 0.15, p = 0.705, partial η² = 0.019	F(1, 8) = 5.52, p = 0.047, partial η² = 0.408
Unpaired two-tailed Student's t-test: tBHP + HupA vs. tBHP + HupA + MK-2206	t(4) = 28.33, p < 0.0001, Cohen's d = 23.1	t(4) = 152.14, p < 0.0001, Cohen's d = 124.2	t(4) = 43.24, p < 0.0001, Cohen's d = 35.3
Sample size	n = 3 per group	n = 3 per group	n = 3 per group

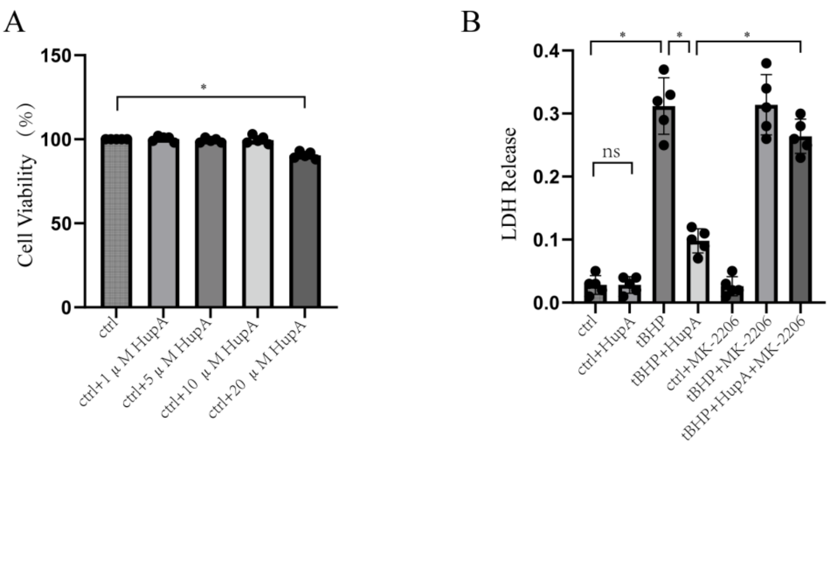

Figure S1 Effects of HupA on cell viability and tBHP-induced injury in H9c2.

(A) H9c2 were treated with increasing concentrations of HupA (1, 5, 10 and 20 μM) for 4 h, and cell viability was determined by CCK8 assay. (B)H9c2 were exposed to tert-butyl hydroperoxide (tBHP in the presence or absence of HupA and the AKT inhibitor MK-2206, and lactate dehydrogenase (LDH) release into the culture medium was measured as an index of membrane integrity and cytotoxicity(n=5). Data are expressed as the mean±SD. *P < 0.05 versus the indicated group; ns, not significant. Each data point represents an independent biological replicate. For LDH release, one-way ANOVA revealed significant overall differences across the seven experimental groups (F= 110.39, $p < 0.0001$, partial $\eta^2 = 0.959$). A two-way factorial ANOVA of the HupA×tBHP design (Ctrl, HupA, tBHP, and tBHP+HupA) demonstrated significant main effects of tBHP ($F(1,16) = 225.39$, $p < 0.0001$, partial $\eta^2 = 0.934$) and HupA ($F(1,16) = 82.37$, $p < 0.0001$, partial $\eta^2 = 0.837$) and a significant HupA×tBHP interaction ($F(1,16) = 82.37$, $p<0.0001$, partial $\eta^2 =0.837$); Tukey's post-hoc test confirmed that HupA markedly attenuated the tBHP-induced increase (tBHP vs. tBHP+HupA, $p < 0.0001$), whereas HupA alone did not differ from the control group (p=1.000). In the MK-2206×tBHP design (Ctrl, MK-2206, tBHP, and tBHP+MK-2206), there was a significant tBHP main effect ($F(1,16) = 344.40$, $p < 0.0001$, partial $\eta^2 = 0.956$), with no MK-2206 main effect ($F(1,16) = 0.00$, $p = 1.000$) and no tBHP×MK-2206 interaction ($F(1,16) = 0.02$, $p = 0.898$; Tukey: tBHP+MK-2206 vs. tBHP, $p = 0.9997$), indicating that MK-2206 alone did not affect the readout or modify tBHP-induced injury. Finally, the effect of MK-2206 on HupA-mediated protection was tested by comparing tBHP+HupA with tBHP+HupA+MK-2206 using an unpaired two-tailed Student's t-test: MK-2206 significantly abolished the protective effect of HupA (0.098±0.019 vs. 0.264±0.027; $t(8) = 11.19$, $p < 0.0001$, Cohen's d = 7.08), although the tBHP+HupA+MK-2206 group remained slightly lower than tBHP alone (one-way ANOVA Tukey, p= 0.168). If the HupA× MK-2206 interaction is additionally assessed by two-way ANOVA limited to the tBHP-challenged groups, it is likewise significant ($F(1,16) = 24.90$, $p = 0.000133$, partial $\eta^2 = 0.609$). *P < 0.05.

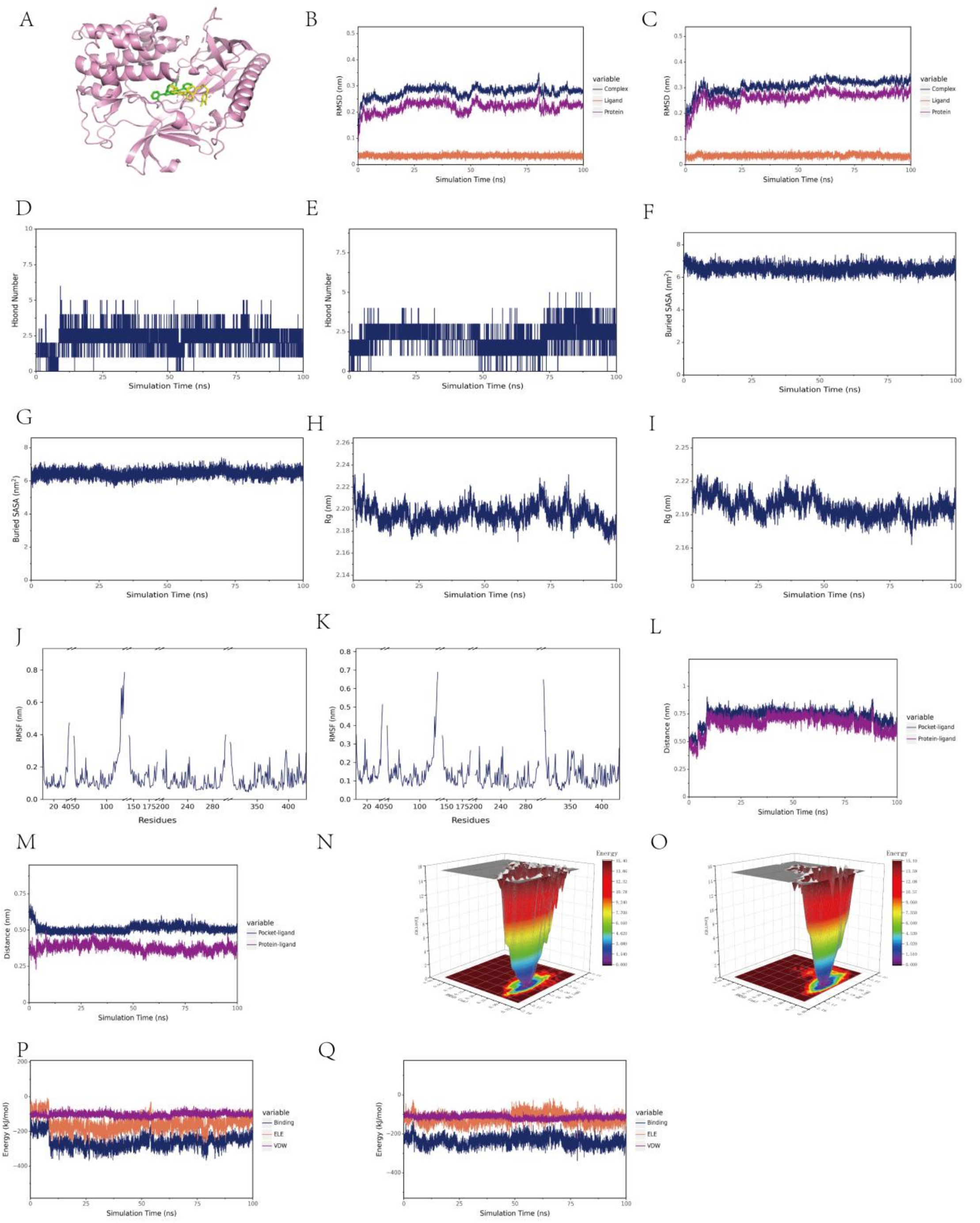

Figure S2 Molecular dynamics simulations of the HupA-AKT1 complex. (A) Redocking validation of AKT1 (PDB: 8UW7). Superposition of the co-crystallized ligand XOO (green) and the top-ranked redocked pose (yellow). The heavy-atom RMSD between the two conformations is 1.60 Å, and the protein backbone is shown in pink. (B, C) Root-mean-square deviation (RMSD) trajectories of the HupA–AKT1 complex, free AKT1, and free HupA over the 100 ns simulations for systems 2 (B) and 3 (C). (D, E) Number of hydrogen bonds formed between HupA and AKT1 during the simulations. (F, G) Buried solvent-accessible surface area (SASA) at the protein–ligand interface. (H, I) Radius of gyration (Rg) of the AKT1–HupA complex. (J, K) Root-mean-square fluctuation (RMSF) profiles of AKT1 residues; axis breaks indicate non-contiguous residue numbering. (L, M) Distances between HupA and the center of the AKT1 binding pocket (pocket–

ligand) and between HupA and the protein mass center (protein–ligand). (N, O) Free-energy landscapes of the HupA–AKT1 complex, projected onto the first two principal components; the color scale denotes the relative free energy (kJ/mol), with the low-energy basin corresponding to the most stable conformational state. (P, Q) MM/PBSA binding free-energy decomposition, showing the total binding free energy (Binding) and its Lennard–Jones (ELJ) and electrostatic (EOW) components .

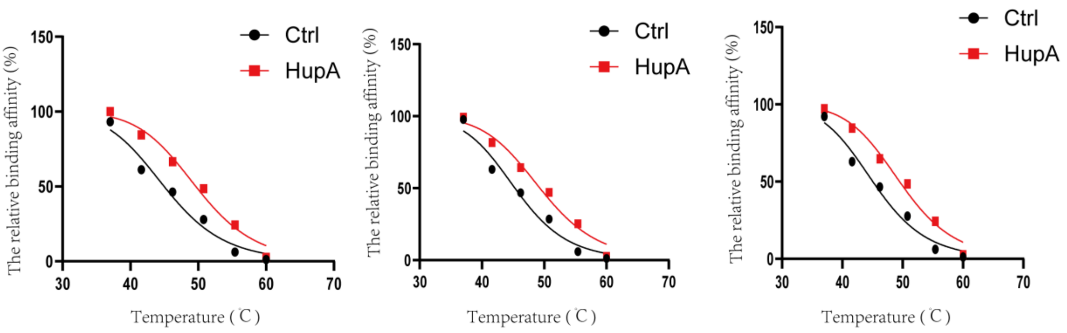

Figure S3 AKT1 thermal melting curves from three independent biological replicates of the cellular thermal shift assay (CETSA). H9c2 cardiomyoblasts were treated with vehicle (Ctrl) or HupA (10 μM) for 3 h; equal aliquots of each cell suspension were then incubated at a temperature gradient (37-60°C) for 3 min, followed by lysis and immunoblotting for AKT1. The soluble AKT1 signal at each temperature, expressed as the percentage of the signal at 37°C within the same replicate, was plotted against temperature and fitted using a four-parameter Boltzmann sigmoidal nonlinear regression model. Each curve represents one independent biological replicate. Individual fitted Tm values were 44.97, 45.30, and 45.05°C (95% CIs: 42.96-46.97, 43.11-47.49, and 43.27-46.82°C) for the control group and 49.64, 49.31, and 49.49°C (95% CIs: 47.91-51.37, 47.40-51.22, and 47.76-51.21°C) for the HupA-treated group, all with excellent goodness of fit (R^2 = 0.97-0.98). The mean fitted Tm was 45.11±0.17°C (95% CI: 44.68-45.53°C) for the control group and 49.48±0.17°C(95% CI: 49.07-49.89°C) for the HupA-treated group, corresponding to a mean thermal shift (ΔTm) of 4.37±0.34°C (range: 4.01-4.67°C across individual replicates). HupA shifted the AKT1 melting curve toward higher temperatures in all three replicates, supporting the reproducibility of the HupA-associated thermal stabilization of AKT1.
